# Supplementary material for: Site-Specific Covalent Labeling of DNA Substrates by an RNA Transglycosylase
Source: J Am Chem Soc. 2023 Mar 29;145(14):8099–106. doi: 10.1021/jacs.3c00861 (PMC10103133; doi:10.1021/jacs.3c00861)
Supplement: Supplementary file 1 — ja3c00861_si_001.pdf [file ja3c00861_si_001.pdf]

Site-specific Covalent Labeling of DNA Substrates by an RNA Transglycosylase

Ember M. Tota and Neal K. Devaraj*

Department of Chemistry and Biochemistry, University of California, San Diego, La Jolla, CA 92093, USA.

Table of Contents

1. **General Information**
2. **Buffers**
3. **Plasmid Design and Sequences**
4. **Oligonucleotide Sequences**
5. **Probe synthesis**
6. **Protein expression and Purification**
7. **TGT labeling reactions**
8. **Cell culture of U2OS cells**
9. **Northern blotting**
10. **Fluorescence in situ hybridization (FISH)**
11. **Supplementary Figures**
12. **MS of PreQ1 probes**
13. **MS of oligo modifications with various probes**
14. **References**

1. **General Methods, Instrument Details and Materials**

All reagents used for buffers and coupling reactions were purchased from Sigma-Aldrich. Cy5-NHS was purchased from Broadpharm, silicon rhodamine-NHS was purchased from Spirochrome, tetramethylrhodamine-NHS was purchased from Lumiprobe, and biotin-NHS, AlexaFluor647-NHS, and AlexaFluor488-NHS were purchased from Thermo Fisher. All oligonucleotides were purchased from Integrated DNA Technologies. Urea PAGE gels were prepared using the 19:1 sequagel system from National Diagnostics, separated using the Mini-Protean system from Biorad, and stained with GelRed from Biotium. All polyacrylamide gels were imaged on a Bio-Rad ChemiDoc-MP gel imager. Gels images were analyzed using ImageJ software from the NIH. U2OS cells were purchased from ATCC and cultured according to supplier recommendations. Fluorescence in situ hybridization (FISH) probe sets were either purchased from Stellaris or designed using the free online tool on the Stellaris website and ordered from IDT. For IR Northern experiments, the RNA was transferred to a positively charged nylon membrane from Invitrogen and crosslinked on a Stratalinker. His purification was conducted using the HisPur Ni-NTA resin from Thermo Fisher and the His-Spin buffers from Zymo. StrepTag purification was carried out using the Strep-Spin kit from Zymo. Protein gels were run using the Mini-Protean gel system and precast gels from Bio-Rad and stained with InstantBlue Coomassie stain from Abcam. All restriction enzymes, bio-reagents, and competent bacterial strains were purchased from New England Biolabs, Promega, or Life Technologies. Absorbance measurements were obtained with a Thermo Scientific Nano-Drop 2000c UV-Vis spectrophotometer. High resolution mass spectrometry (HRMS) of the small molecule probes and mass spectrometry (MS) of the oligonucleotide samples were collected by the UCSD Department of Chemistry and Biochemistry Molecular Mass Spectroscopy Facility on an Agilent 6230 time-of-flight mass spectrometer (TOFMS) with JetStream electrospray ionization source (ESI).

2. **Buffers**

His lysis and wash buffer: 50 mM sodium phosphate buffer pH 7.7, 300 mM sodium chloride, 50 mM imidazole, 0.03 % Triton X-100. (Zymo P2003-4; supplemented with 400 µM PMSF)

His elution buffer: 50 mM sodium phosphate buffer pH 7.7, 300 mM sodium chloride, 250 mM imidazole. (Zymo P2003-5; supplemented with 400 µM PMSF)

TGT storage buffer: 25 mM HEPES, pH 7.5, 100 mM NaCl, 100 µM PMSF.

TGT reaction buffer 1X: 100 mM HEPES, pH 7.3, 5 mM DTT, and 20 mM MgCl2

Northern hybridization buffer: NorthernMax Prehybridization/Hybridization Buffer (Invitrogen AM8677)

Northern wash buffer: Northern Max Low Stringency wash buffer (Invitrogen AM8673)

FISH buffer A: 1:7:2 deionized formamide:water:Stellaris Buffer A (Biosearch Technologies SMF-WA1-60)

FISH buffer B: Stellaris Buffer B (Biosearch Technologies SMF-WB1-20)

FISH hybridization buffer: 9:1 Stellaris Hybridization Buffer:deionized formamide (Biosearch Technologies SMF-HB1-10)

In vitro transcription buffer: 40 mM Tris pH 7.5, 5 mM DTT, 25 mM MgCl2, 2 mM spermidine

3. **Plasmid Design and Sequences**

Plasmid Design: A second purification tag was added to the TGT-His construct previously reported[2] (Addgene 138201) using the online NEBase changer tool and Q5 site directed mutagenesis kit (NEB E0554S). Two constructs with either an N- or C-terminal Strep purification tag were assembled and used interchangeably. Purification tag sequences are bolded and underlined below. The constructs were transformed into high efficiency 5-alpha competent *E. coli* cells (NEB C2987H), plated, and grown overnight on an LB agar plate containing Kanamycin. The next day, individual colonies were picked, expanded in a 10 mL culture, mini prepped (Biomiga PD1213), and the resulting plasmids sequenced by Eton Biosciences.

TGT-Strep-His Coding Sequence: ATGAAATTTGAACTGGACACCACCGACGGTCGCGCACGCCG
TGGCCGCCTGGTCTTTGATCGTGGCGTAGTGGAAACGCCTTGTTTTATGCCTGTTGGCACCTACGGCACC
GTAAAAGGGATGACGCCGGAAGAAGTTGAAGCCACTGGCGCGCAAATTATCCTCGGCAACACCTTCCAC
CTGTGGCTGCGCCCGGGCCAGGAAATCATGAAACTGCACGGCGATCTGCACGATTTTATGCAGTGGAA
GGGGCCGATCCTCACCGACTCCGGCGGCTTCCAGGTCTTCAGCCTTGGCGATATTCGTAAAATCACCGA
ACAGGGCGTGCACTTCCGTAACCCGATCAACGGCGATCCGATTTTCCTCGATCCTGAAAAATCAATGGA
GATTCAGTACGATCTTGGTTCGGATATCGTCATGATCTTTGATGAGTGTACGCCGTATCCTGCTGACTGG
GATTACGCAAAACGCTCCATGGAGATGTCTCTGCGTTGGGCGAAGCGTAGCCGTGAGCGTTTTGACAGT
CTCGGAAACAAAAATGCGCTGTTTGGTATCATCCAGGGCAGCGTTTACGAAGATTTACGTGATATTTCTG
TTAAAGGTCTGGTAGATATCGGTTTTGATGGCTACGCTGTCGGCGGTCTGGCTGTGGGTGAGCCGAAAG
CAGATATGCACCGCATTCTGGAGCATGTATGCCCGCAAATTCCGGCAGACAAACCGCGTTACCTGATGG
GCGTTGGTAAACCAGAAGACCTGGTTGAAGGCGTACGTCGTGGTATCGATATGTTTGACTGCGTAATGC
CAACCCGCAACGCCCGAAATGGTCATTTGTTCGTGACCGATGGCGTGGTGAAAATCCGCAATGCGAAGT
ATAAGAGCGATACTGGCCCACTCGATCCTGAGTGTGATTGCTACACCTGTCGCAATTATTCACGCGCTTA
CTTGCATCATCTTGACCGTTGCAACGAAATATTAGGCGCGCGACTCAACACCATTCATAACCTTCGTTACT
ACCAGCGTTTGATGGCGGGTTTACGCAAGGCTATTGAAGAGGGTAAATTAGAGAGCTTCGTAACTGATT
TTTACCAGCGTCAGGGGCGAGAAGTACCACCTTTGAACGTTGATGGC**TGGAGCCACCCGCAGTTCGAA**
AAACTCGAG**CACCATCACCACCATCAC**TAA

Strep-TGT-His Coding Sequence: ATG**TGGAGCCATCCGCAGTTTGAAAAA**GGCAAATTTGAACT
GGACACCACCGACGGTCGCGCACGCCGTGGCCGCCTGGTCTTTGATCGTGGCGTAGTGGAAACGCCTTG
TTTTATGCCTGTTGGCACCTACGGCACCGTAAAAGGGATGACGCCGGAAGAAGTTGAAGCCACTGGCGC
GCAAATTATCCTCGGCAACACCTTCCACCTGTGGCTGCGCCCGGGCCAGGAAATCATGAAACTGCACGG
CGATCTGCACGATTTTATGCAGTGGAAGGGGCCGATCCTCACCGACTCCGGCGGCTTCCAGGTCTTCAG
CCTTGGCGATATTCGTAAAATCACCGAACAGGGCGTGCACTTCCGTAACCCGATCAACGGCGATCCGAT
TTTCCTCGATCCTGAAAAATCAATGGAGATTCAGTACGATCTTGGTTCGGATATCGTCATGATCTTTGATG
AGTGTACGCCGTATCCTGCTGACTGGGATTACGCAAAACGCTCCATGGAGATGTCTCTGCGTTGGGCGA
AGCGTAGCCGTGAGCGTTTTGACAGTCTCGGAAACAAAAATGCGCTGTTTGGTATCATCCAGGGCAGCG
TTTACGAAGATTTACGTGATATTTCTGTTAAAGGTCTGGTAGATATCGGTTTTGATGGCTACGCTGTCGG
CGGTCTGGCTGTGGGTGAGCCGAAAGCAGATATGCACCGCATTCTGGAGCATGTATGCCCGCAAATTCC
GGCAGACAAACCGCGTTACCTGATGGGCGTTGGTAAACCAGAAGACCTGGTTGAAGGCGTACGTCGTG
GTATCGATATGTTTGACTGCGTAATGCCAACCCGCAACGCCCGAAATGGTCATTTGTTCGTGACCGATGG
CGTGGTGAAAATCCGCAATGCGAAGTATAAGAGCGATACTGGCCCACTCGATCCTGAGTGTGATTGCTA
CACCTGTCGCAATTATTCACGCGCTTACTTGCATCATCTTGACCGTTGCAACGAAATATTAGGCGCGCGA
CTCAACACCATTCATAACCTTCGTTACTACCAGCGTTTGATGGCGGGTTTACGCAAGGCTATTGAAGAGG
GTAAATTAGAGAGCTTCGTAACTGATTTTTACCAGCGTCAGGGGCGAGAAGTACCACCTTTGAACGTTG
AT**CACCATCACCACCATCAC**TAA

4. **Oligonucleotides**

Key hairpins

Table S1. Key hairpins referenced in this work. RNA hairpins are shown in blue. DNA hairpins are shown in black.

Design	Hairpin	Sequence
original hairpins	ECY-A1	GCAGACUGUAAAUCUGC
	ECYMH	GGGAGCAGACUGUAAAUCUGCUCCC
mini helix mutants	ECYMH-rTGrT	GGGAGCAGACrTGrTAAArTCrTGCrTCCC
	dECYMH	GGGAGCAGACTGTAAATCTGCTCCC
	dECYMH-dUGdU	GGGAGCAGACdUGdUAAATCTGCTCCC
	dECYMH-dUGT	GGGAGCAGACdUGTAAATCTGCTCCC
	dECYMH-TGdU	GGGAGCAGACTGdUAAATCTGCTCCC
tRNA analogs	dECY-0 (dECY-A1)	GCAGACTGTAAATCTGC
	dECH-0	CTGGATTGTGATTCCAG
tRNA chimera	dECH-dECD	CTGGACTGTCACTCCAG
top dECH-9 loop mutants	dECH-10	CTGGATTGTCCTTCCAG
	dECH-18	CTGGACTGTCCCTCCAG
	dECH-24	CTGGACTGTTCCTCCAG
	dECH-26	CTGGATTGTCCCTCCAG
	dECH-32	CTGGATTGTTCCTCCAG
	dECH-34	CTGGACTGTCCTTCCAG
ΔC mutant	dECH-10 ΔC	CTGGATTCTCCTTCCAG
top stem sequence mutants dECH-10 loop	162_ala2_dECH-10	CCTGCTTGTCCTGCAGG
	164_arg2_dECH-10	CTCGGTTGTCCTCCGAG
	165_arg3_dECH-10	CTGCCTTGTCCTGGCAG
	170_gln1_dECH-10	CCGGATTGTCCTTCCGG
	171_gln2_dECH-10	CCGGTTTGTCCTACCGG
	172_glu1_dECH-10	CCGCCTTGTCCTGGCGG
	174_gly2_dECH-10	TCAGCTTGTCCTGCTGA
	175_gly3_dECH-10	CGACCTTGTCCTGGTCG
	176_his1_dECH-10	CTGGATTGTCCTTCCAG
	177_ile1_dECH-10	CACCCTTGTCCTGGGTG
	180_leu3_dECH-10	CTACCTTGTCCTGGTAG
	194_ser3_dECH-10	CTCCCTTGTCCTGGGAG
	197_thr2_dECH-10	TCGCATTGTCCTTGCGA
	198_thr3_dECH-10	CGCATTTGTCCTATGCG
	203_val1_dECH-10	CCACCTTGTCCTGGTGG
	204_val2_dECH-10	CCTCCTTGTCCTGGAGG

Analog Hairpins

Table S2. Analog hairpins based on known constructs. RNA hairpins are shown in blue. DNA hairpins are shown in black. Key hairpins are also included in this table.

Design	Hairpin	Sequence
original hairpins	rECY-A1	GCAGACUGUAAAUCUGC
	rECYMH	GGGAGCAGACUGUAAAUCUGCUCCC
mini helix mutants	rECYMH-rTGrT	GGGAGCAGACrTGrTAAArTCrTGCrTCCC
	dECYMH	GGGAGCAGACTGTAAATCTGCTCCC
	dECYMH-dUGdU	GGGAGCAGACdUGdUAAATCTGCTCCC
	dECYMH-dUGT	GGGAGCAGACdUGTAAATCTGCTCCC
	dECYMH-TGdU	GGGAGCAGACTGdUAAATCTGCTCCC
tRNA analogs	dECY-0 (dECY-A1)	GCAGACTGTAAATCTGC
	dECH-0	CTGGATTGTGATTCCAG
	dECD-0	CCTGCCTGTCACGCAGG
	dECN-0	GCGGACTGTTAATCCGT
tRNA chimeras	dECD-dECH	CCTGCTTGTGATGCAGG
	dECD-dECY	CCTGCCTGTAAAGCAGG
	dECD-dECN	CCTGCCTGTTAAGCAGG
	dECN-dECH	GCGGATTGTGATTCCGT
	dECN-dECY	GCGGACTGTAAATCCGT
	dECN-dECD	GCGGACTGTCACTCCGT
	dECY-dECH	GCAGATTGTGATTCTGC
	dECY-dECN	GCAGACTGTTAATCTGC
	dECY-dECD	GCAGACTGTCACTCTGC
	dECH-dECY	CTGGACTGTAAATCCAG
	dECH-dECN	CTGGACTGTTAATCCAG
	dECH-dECD	CTGGACTGTCACTCCAG

Mutant Hairpins

Table S3. Loop and stem mutant hairpins based on anticodon arms of tRNAs. Redundant hairpins are group by color. Key hairpins are also included in these tables.

	Hairpin	Sequence
dECH mutants	dECH-1	CTCGATTGTGATTCGAG
	dECH-2	CTAGATTGTGATTCTAG
	**dECH-3**	**CTGGATTGTTATTCCAG**
	dECH-4	CTGCATTGTGATTGCAG
	dECH-5	CTGAATTGTGATTTCAG
	dECH-6	GTGGATTGTGATTCCAC
	dECH-7	GTGGATTGTGATTCCAT
	dECH-8	ATGGATTGTGATTCCAT
	dECH-9	CTGGATTGTCATTCCAG
	dECH-10	CTGGATTGTCCTTCCAG
	dECH-11	CTGGATTGTTTTTCCAG
	**dECH-12**	**CTGGATTGTTATTCCAG**
	dECH-13	CTGGATTGTCTTTCCAG
	dECH-14	CTGGATTGTCGTTCCAG
	dECH-15	CTGGATTGTTGTTCCAG
	dECH-16	CTGGATTGTTCTTCCAG
	dECH-17	CTGGACTGTCACTCCAG
	dECH-18	CTGGACTGTCCCTCCAG
	dECH-19	CTGGACTGTTTCTCCAG
	dECH-20	CTGGACTGTTACTCCAG
	dECH-21	CTGGACTGTCTCTCCAG
	dECH-22	CTGGACTGTCGCTCCAG
	dECH-23	CTGGACTGTTGCTCCAG
	dECH-24	CTGGACTGTTCCTCCAG
	dECH-25	CTGGATTGTCACTCCAG
	dECH-26	CTGGATTGTCCCTCCAG
	dECH-27	CTGGATTGTTTCTCCAG
	dECH-28	CTGGATTGTTACTCCAG
	dECH-29	CTGGATTGTCTCTCCAG
	dECH-30	CTGGATTGTCGCTCCAG
	dECH-31	CTGGATTGTTGCTCCAG
	dECH-32	CTGGATTGTTCCTCCAG
	dECH-33	CTGGACTGTCATTCCAG
	dECH-34	CTGGACTGTCCTTCCAG
	dECH-35	CTGGACTGTTTTTCCAG
	dECH-36	CTGGACTGTTATTCCAG
	dECH-37	CTGGACTGTCTTTCCAG
	dECH-38	CTGGACTGTCGTTCCAG
	dECH-39	CTGGACTGTTGTTCCAG
	dECH-40	CTGGACTGTTCTTCCAG

	Hairpin	Sequence
dECH-10 loop/tRNA stem mutants	161_ala1_dECH-10	CTTGCTTGTCCTGCAAG
	**162_ala2_dECH-10**	**CCTGCTTGTCCTGCAGG**
	163_arg1_dECH-10	AGCCCTTGTCCTGGGCT
	164_arg2_dECH-10	CTCGGTTGTCCTCCGAG
	165_arg3_dECH-10	CTGCCTTGTCCTGGCAG
	166_arg4_dECH-10	ACGACTTGTCCTGTCGT
	**167_asn1_dECH-10**	**GCGGATTGTCCTTCCGT**
	**168_asp1_dECH-10**	**CCTGCTTGTCCTGCAGG**
	**169_cys1_dECH-10**	**GCGGATTGTCCTTCCGT**
	170_gln1_dECH-10	CCGGATTGTCCTTCCGG
	**171_gln2_dECH-10**	**CCGGTTTGTCCTACCGG**
	172_glu1_dECH-10	CCGCCTTGTCCTGGCGG
	173_gly1_dECH-10	AGAGCTTGTCCTGCTCT
	174_gly2_dECH-10	TCAGCTTGTCCTGCTGA
	175_gly3_dECH-10	CGACCTTGTCCTGGTCG
	176_his1_dECH-10	CTGGATTGTCCTTCCAG
	**177_ile1_dECH-10**	**CACCCTTGTCCTGGGTG**
	178_leu1_dECH-10	GTTGATTGTCCTTCAAC
	179_leu2_dECH-10	CTAGCTTGTCCTGTTAG
	180_leu3_dECH-10	CTACCTTGTCCTGGTAG
	181_leu4_dECH-10	CCAGATTGTCCTTCTGG
	182_leu5_dECH-10	AGGGATTGTCCTTCCCT
	183_lys1_dECH-10	GTTGATTGTCCTTCAAT
	**184_met1_dECH-10**	**GGCGATTGTCCTTCGCT**
	185_met2_dECH-10	CATCATTGTCCTTGATG
	**186_met3_dECH-10**	**GGCGATTGTCCTTCGCT**
	187_met4_dECH-10	TCGGGTTGTCCTCCCGA
	188_phe1_dECH-10	GGGGATTGTCCTTCCCC
	189_pro1_dECH-10	ACTGGTTGTCCTCCAGT
	190_pro2_dECH-10	CCGTCTTGTCCTGTCGG
	191_pro3_dECH-10	CTTCGTTGTCCTCGAGG
	**192_ser1_dECH-10**	**CCGGTTTGTCCTACCGG**
	**193_ser2_dECH-10**	**CCGGTTTGTCCTACCGG**
	194_ser3_dECH-10	CTCCCTTGTCCTGGGAG
	195_ser4_dECH-10	CACGCTTGTCCTGTGTG
	**196_thr1_dECH-10**	**CACCCTTGTCCTGGGTG**
	197_thr2_dECH-10	TCGCATTGTCCTTGCGA
	198_thr3_dECH-10	CGCATTTGTCCTATGCG
	199_thr4_dECH-10	ACTGATTGTCCTTCAGT
	**200_thr5_dECH-10**	**CACCCTTGTCCTGGGTG**
	**201_trp1_dECH-10**	**CCGGTTTGTCCTACCGG**
	202_tyr1_dECH-10	GCAGATTGTCCTTCTGC
	203_val1_dECH-10	CCACCTTGTCCTGGTGG
	204_val2_dECH-10	CCTCCTTGTCCTGGAGG

Oligo Extension Hairpins

Table S4. Labeling of oligos containing one or two recognition hairpins. DNA-TAG recognition hairpins are underlined.

**Oligo Extension Hairpins**		
**Design**	**Hairpin**	**Sequence**
single hairpin	His M10 T2 int	ACCATCATTTTCATATCCTCCActggaTTGTCCTtccagACCACCATCATTTGCAATGA
	His M10 T2 3'	ACCATCATTTTCATATCCTCCAACCACCATCATTTGCAATGActggaTTGTCCTtccag
	His M10 T2 5'	ctggaTTGTCCTtccagACCATCATTTTCATATCCTCCAACCACCATCATTTGCAATGA
double hairpin	5/3_176/177_**GG**	ctggaTT**G**TCCTtccagCATCATTTTCATATCCTCCAcacccTT**G**TCCTgggtg
	5/3_176/177_**GC**	ctggaTT**G**TCCTtccagCATCATTTTCATATCCTCCAcacccTT**C**TCCTgggtg
	5/3_176/177_**CG**	ctggaTT**C**TCCTtccagCATCATTTTCATATCCTCCAcacccTT**G**TCCTgggtg
	5/5_176/177_**GG**	ctggaTT**G**TCCTtccagTTTcacccTT**G**TCCTgggtgCATCATTTTCATATCCTCCA
	5/5_176/177_**GC**	ctggaTT**G**TCCTtccagTTTcacccTT**C**TCCTgggtgCATCATTTTCATATCCTCCA
	5/5_176/177_**CG**	ctggaTT**C**TCCTtccagTTTcacccTT**G**TCCTgggtgCATCATTTTCATATCCTCCA
	3/3_176/177_**GG**	CATCATTTTCATATCCTCCActggaTT**G**TCCTtccagTTTcacccTT**G**TCCTgggtg
	3/3_176/177_**GC**	CATCATTTTCATATCCTCCActggaTT**G**TCCTtccagTTTcacccTT**C**TCCTgggtg
	3/3_176/177_**CG**	CATCATTTTCATATCCTCCActggaTT**C**TCCTtccagTTTcacccTT**G**TCCTgggtg

In vitro transcription (IVT) of ECYMH-rTGrT. Forward and reverse template constructs were ordered from IDT and annealed (Table S5). The transcription was carried out with the following conditions using T7 RNA polymerase prepared as previously described.[1] 1X T7 RNAP Buffer, 5 mM MeUTP, 5 mM ATP, 5 mM CTP, 9 mM GTP, 5 mM annealed template, 0.004 U/µL PPTase, 0.05% Triton X, and 0.15 µg/µL T7 RNAP and incubated at 4˚C for 4 hours. Subsequently, 1.2 µL 100 mM $CaCl_2$ and 1 µL Turbo DNAse (Thermo Fisher AM2238) were added to a 100 µL IVT reaction and incubated at 37˚C for 20 minutes. The RNA product was then purified by ethanol precipitation and the transcript analyzed by urea PAGE.

Table S5. IVT template for rECYMH-rTGrT. The T7 promoter sequence is underlined, and the hairpin sequence is in bold.

**Construct**	**Sequence**
**rECYMH-rTGrT FWD**	GAAATTAATACGACTCACTATA**GGGAGCAGACTGTAAATCTGCTCCC**
**rECYMH-rTGrT REV**	**GGGAGCAGATTTACAGTCTGCTCCC**TATAGTGAGTCGTATTAATTTC

5. **Probe Synthesis**

All probes were synthesized as described previously by removing a tert-butyloxycarbonyl (boc) protecting group from a preQ1 derivative and coupling it to commercially available NHS functionalized reporters.[1,2] Briefly, the boc protected starting material was deprotected in 10% TFA/DCM for one hour. The TFA was neutralized 3 times with 10% TEA in DCM, rotoevaporating the solution to near dryness between each wash. The crude oil product was taken to the next step where it was dissolved in dry DMF followed by the dropwise addition of TEA. Each NHS probe was dissolved in dry DMF and added to the reaction dropwise with stirring. The reaction was allowed to proceed for 2 hours at room temperature, the DMF evaporated, and the product purified by HPLC. The final products were confirmed by LCMS.

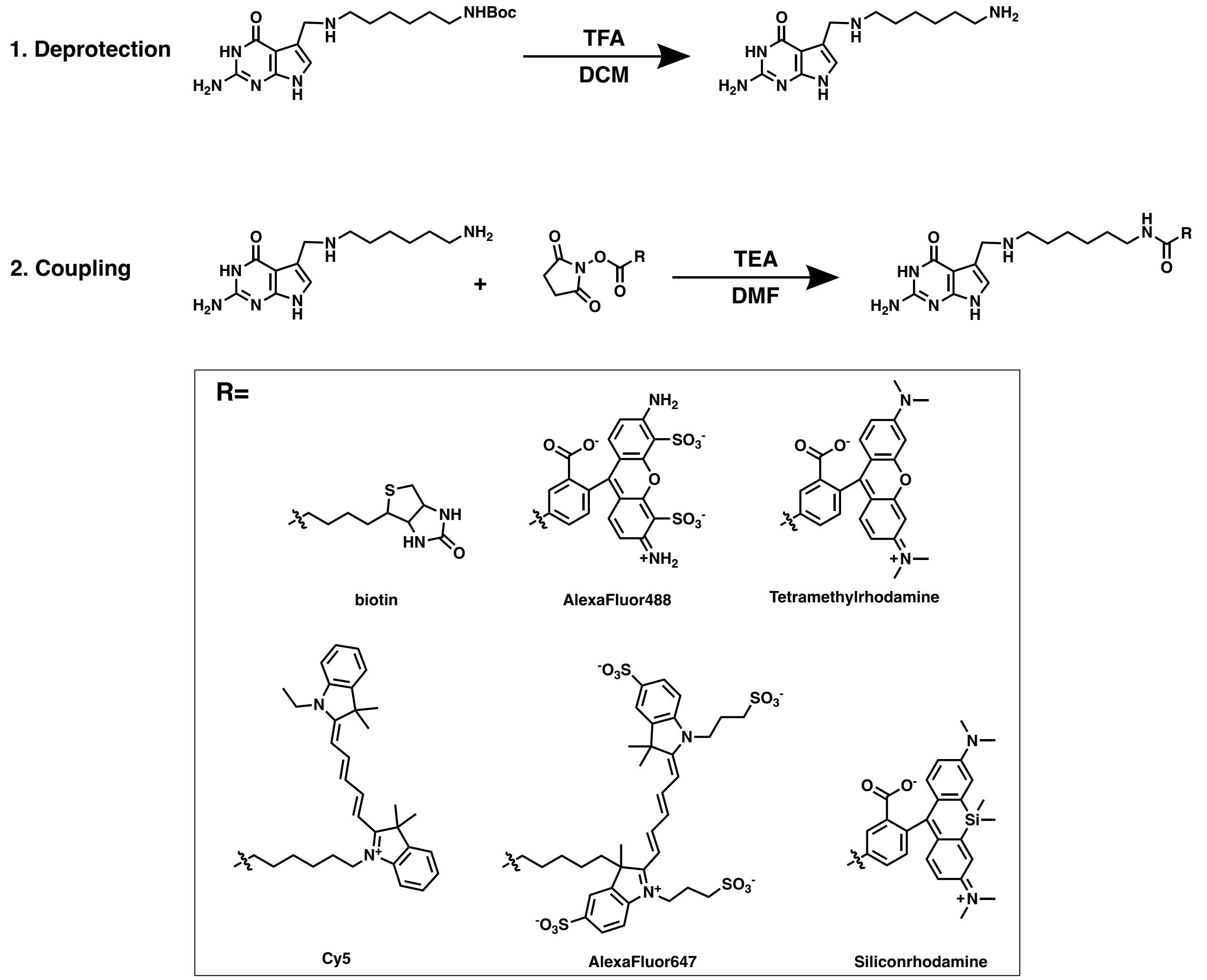

Scheme S1. Synthesis of preQ1 probes with commercially purchased preQ1-C_6NHBoc.

6. **Protein Expression and Purification**

Expression of TGT *E. coli* TGT was expressed as previously described from the plasmid described in section 3.[2] Briefly, the plasmid carrying TGT was transformed into BL21(DE3) Competent *E. coli* cells (NEB C2527) and a starter culture was grown overnight. Approximately 5 mL of starter culture was transferred into 200 mL of kanamycin containing LB and allowed to grow to an optical density of ~0.6. At this point, expression was induced with by adding IPTG to a final concentration of 1 mM and shaken at 37˚C for 4 hours. The bacteria were pelleted by centrifugation (6,000xg for 20 minutes at 4˚C). Cell pellets were either resuspended and purified immediately or stored at -20˚C if desired.

Purification of TGT A dual purification (His and streptactin) protocol was used to increase purity of the enzyme. 10µL aliquots were taken at each step for analysis via 4-20% SDS PAGE.

His purification was carried out using HisPur^{TM} Ni-NTA Resin spin columns (Thermo Fisher 88224) and His purification buffers (Zymo P2003-4; P2003-5). The cell pellet was resuspended in 2 mL total of lysis buffer and sonicated on ice using the following settings: 4 cycles (30 sec on / 2 min off); output control = 4; duty cycle% = 30-40. The lysate was centrifuged at 10,000xg for 30 min at 4˚C to remove cellular debris. In tandem, the resin from the spin column was equilibrated 2 times with 400 µL of lysis buffer. The supernatant of the centrifuged cell lysate was transferred to the capped spin column and incubated for at least one hour at 4˚C with end over end mixing. The resin was then washed and the protein eluted according to the HisPur manual. The lysis and wash buffer are the same as it was noted that a higher imidazole concentration during binding reduced nonspecific carry over into the final elutions. The elution was taken directly to streptactin purification.

Streptactin purification was carried out using the Strep-Spin Protein Miniprep Kit (Zymo P2004). It should be noted that the protein sample incubation time was increased to 30-60 minutes at 4˚C to increase yield. After elution from the streptactin column, the enzyme prep was dialyzed against TGT storage buffer to remove biotin and concentrated using a 30 kDa cut off spin filter (Millipore UFC503096). Enzyme concentration was determined using a BCA assay kit (Thermo Fisher 23227). The final protein was aliquoted in 20 µL fractions and stored at -80˚C.

7. **TGT labeling and analysis**

TGT labeling reaction: TGT modification of DNA and RNA substrates was carried out at 37˚C for 4 hours in a thermocycler with a heated lid[1,2] using the following reaction conditions: 10 µM enzyme, 5 µM nucleic acid substrate, and 50 µM of preQ1-X (various preQ1 based probes), 1X TGT reaction buffer, and 1-unit RNAsin from Promega for RNA substrates. Components were mixed together, gently vortexed, briefly spun down, and incubated on a thermocycler. For labeling reactions of constructs containing 2 hairpins, the small molecule substrate concentration was doubled. For analysis purposes, reactions were not purified before PAGE, which did not affect the gel shift.

Labeled oligo purification: For the experiments where it was necessary to purify the labeled construct, an oligo clean and concentrator kit (Zymo D4060) was used, and the product eluted in pure water.

Urea PAGE: Urea PAGE was the primary method of analysis to determine TGT activity toward specific substrates. For all gels, we used the 19:1 Acrylamide:Bisacrylamide SequaGel® UreaGel System (National Diagnostics EC-833) and the Mini-PROTEAN Tetra Vertical Electrophoresis Cell from Bio- Rad. Briefly, 1.5 mm plates assembled on the Bio-Rad gel casting stand, 10 mL of the desired percentage gel was freshly prepared in a 15 mL centrifuge tube, 14 µL of TEMED was added and gently mixed by inverting the tube, followed by 80 µL of 10% ammonium per sulfate. After briefly mixing, the solution is poured between the plates, a comb inserted, and allowed to solidify (~20-30 min). All oligo samples were prepared for gel electrophoresis using 2X RNA loading dye (NEB B0363S). For all constructs, 150-200 ng of the oligonucleotide was loaded onto the gel for visualization. The solutions were denatured at 98˚C for 5 minutes on a thermocycler. The electrophoresis cassette was assembled, and the chamber filled with 1X TBE. After thorough washing of the wells to remove leached urea, the samples were loaded into the wells and the gel was run for 10 min at 100V then 90 min at 200V. Afterward, the gel was stained with gel red in TBE (1:1000 dilution; Biotium 41011) for ~5 min and imaged on a Bio-Rad ChemiDoc gel imager system.

Gel Shift Analysis: Images were exported for analysis form the ChemiDoc software and analyzed qualitatively by eye. Successful labeling is indicated by an upward shift in the product lane due to an increase in molecular weight of the labeled species. In the cases where a quantitative measurement was desired, the bands were analyzed via densitometry using ImageJ. Briefly, the percent signal of each band compared to the total in the control lane was calculated.

8. **Cell Culture of U2OS Cells**

For Northern and FISH experiments, we used U2OS cells, a human osteosarcoma derived line. The cells were ordered from ATCC (HTB-96), thawed, expanded, and stocks stored at -80˚C. Cells were cultured according to ATCC guidelines in T25 flasks. Complete growth medium containing DMEM (Gibco 11995065), 10% FBS, and 1% pen/strep (Gibco 15070063) was used to maintain and culture the cells. The cells were passaged ~3 times per week at a 1:6 sub cultivation ratio when they reached ~80% confluency. To passage the cells, they were washed 1X with HBSS (Gibco 14025076) and treated with TrypLE Express (Gibco 12604013) at 37˚C until fully lifted. The trypsin was quenched by adding an equal volume of complete medium and the necessary volume of cells taken into a new flask. For experiments, cells were seeded in a black glass bottom 24 well plate at a 1:6 ratio calculated to scale with the area of the plate.

9. **Northern blot**

DNA was extracted from U2OS cells and the U6 sequence amplified with a primer set that includes the T7 promoter sequence. This amplified product was purified and used as a template for in vitro transcription. The IVT RNA was purified by ethanol precipitation and analyzed via urea PAGE. Total RNA was extracted from U2OS cells. The U6 PCR amplified template, IVT U6 RNA, and total extracted U2OS RNA were analyzed via gel electrophoresis.

Table S6. Relevant sequences for U6 RNA northern blot. The target and antisense sequences are underlined and the DNA-TAG recognition hairpin is in bold.

Construct	Sequence
**U6 RNA**	GUGCUCGCUUCGGCAGCACAUAUACUAAAAUUGGAACGAUACAGAGAAGAUUAGCAUGGCCCCUGCGCAAGGAUGACACGCAAAUUCGUGAAGCGUUCCAUAUUUU
**5′ dECH-10 U6 probe**	**CTGGATTGTCCTTCCAG**TTTGCAGGGGCCATGCTAATCTTCTCTGTATCG
**T7 U6 amp fwd**	ATACGACTCACTATAGGTGCTCGCTTCGGCAG
**T7 U6 amp rev**	AAAATATGGAACGCTTCACGAATTTGCGTGTC

U6 RNA Template Preparation Genomic DNA was extracted from U2OS cells using the Quick-DNA Miniprep Plus Kit (Zymo D4068). The U6 RNA IVT template was PCR amplified from ~10 ng of the isolated genomic DNA using Q5® Hot Start High-Fidelity 2X Master Mix (NEB M0494). The PCR product was verified by gel electrophoresis, purified using a DNA clean and concentrator kit (Zymo D4029), and used for the IVT of U6 RNA.

IVT (In vitro Transcription) The U6 RNA transcript was *in vitro* transcribed with the following conditions using T7 RNA polymerase prepared as previously described:[1] 1X T7 RNAP Buffer, 5 mM UTP, 5 mM ATP, 5 mM CTP, 9 mM GTP, 5 mM Annealed template, 0.004 U/µL PPTase, 0.05% Triton X, and 0.15 µg/µL T7 RNAP and incubated at 4˚C for 4 hours. Subsequently, 1.2 µL 100 mM CaCl2 and 1 µL Turbo DNAse (Thermo Fisher AM2238) were added to a 100 µL IVT reaction, incubated at 37˚C for 20 minutes. The RNA product was then purified by ethanol precipitation and the transcript analyzed by urea PAGE.

RNA Extraction Total RNA was extracted from U2OS cells using the Direct-zol RNA Miniprep Kit (Zymo R2053) and analyzed by urea PAGE.

Northern Blot Dilution series of in vitro transcribed U6 RNA or U2OS total RNA were run on a 15% urea PAGE gel as described above. The RNA was transferred to a positively charged nylon membrane (Invitrogen AM10100) using the Mini Trans-Blot cell system from Bio-Rad. The transfer was done at room temperature and run at 200 mA for 30 min in 0.5X TBE. The membrane was washed in 10X SSC buffer for 10 minutes with rocking and crosslinked twice on a Stratalinker using the auto setting. The membrane was then incubated with the 5′ SiR labeled U6 antisense probe in Northern Hybridization Buffer (10 picomolar; 2hr 37˚C), washed 2X for 5 minutes each with Northern Max Low Stringency wash buffer, and imaged on a fluorescent gel scanner. The signal was quantified via densitometry using ImageJ and analyzed for linear fit.

10. **RNA Fluorescence in situ Hybridization (FISH)**

U2OS cells were seeded in glass bottom black 24 well plates at a ratio of 1:6 and allowed to grow to ~80% confluency. Cells were washed with HBSS and fixed in 4% para formaldehyde (PFA) for 10 minutes at room temperature. PFA was aspirated, the cells were washed twice with HBSS, and permeabilized with 70% ethanol overnight at 4˚C. The ethanol solution was removed, and the cells washed 2X with HBSS. FISH buffer A was added and let sit for 5 minutes. Fluorescent probe sets were prepared in hybridization buffer with a final concentration of 125 nM and added to the wells. Parafilm was placed over the wells and around the lid of the plate to minimize evaporation and the plate incubated at 37˚C overnight in the dark. The next morning, the probe solution was aspirated, and the cells washed in FISH buffer A for 30 min at 37˚C in the dark. FISH buffer A was then aspirated from the cells, a solution of 5 ng/mL DAPI in FISH buffer A was added, and the plate was placed at 37˚C for 30 minutes. The DAPI solution was aspirated, and the cells washed in FISH buffer B for 5 minutes. FISH buffer B was aspirated and Vectashield (Vector Laboratories H-1000-10) added to the wells. The cells were imaged on a confocal microscope. All images were window leveled and analyzed visually using ImageJ. For wells with multiple probe sets, equal amounts of each set were added to a final total concentration of 125 nM.

Table S7. MALAT 1 single label probe set.

Probe	Sequence
**MALAT1_1_176/177_3'3'_GC**	GCCCTTCTATTGGTATTAATTTTCTGGATTGTCCTTCCAGTTTCACCCTTCTCCTGGGTG
**MALAT1_2_176/177_3'3'_GC**	CTCCCAATTAATCTTTCCATTTTCTGGATTGTCCTTCCAGTTTCACCCTTCTCCTGGGTG
**MALAT1_3_176/177_3'3'_GC**	TCTCCAAATTGTTTCATCCTTTTCTGGATTGTCCTTCCAGTTTCACCCTTCTCCTGGGTG
**MALAT1_4_176/177_3'3'_GC**	TTAGCTTTTTGTTTCCTAGCTTTCTGGATTGTCCTTCCAGTTTCACCCTTCTCCTGGGTG
**MALAT1_5_176/177_3'3'_GC**	ATCTTCTCAAGCTTTACCTTTTTCTGGATTGTCCTTCCAGTTTCACCCTTCTCCTGGGTG
**MALAT1_6_176/177_3'3'_GC**	ACTATATTTAAGGCCTTCCATTTCTGGATTGTCCTTCCAGTTTCACCCTTCTCCTGGGTG
**MALAT1_7_176/177_3'3'_GC**	TACTTCCGTTACGAAAGTCCTTTCTGGATTGTCCTTCCAGTTTCACCCTTCTCCTGGGTG
**MALAT1_8_176/177_3'3'_GC**	CTGGGTCAGCTGTCAATTAATTTCTGGATTGTCCTTCCAGTTTCACCCTTCTCCTGGGTG
**MALAT1_9_176/177_3'3'_GC**	TCAGTCCTAGCTTCATCAAATTTCTGGATTGTCCTTCCAGTTTCACCCTTCTCCTGGGTG
**MALAT1_10_176/177_3'3'_GC**	AACAACATATTGCCGACCTCTTTCTGGATTGTCCTTCCAGTTTCACCCTTCTCCTGGGTG
**MALAT1_11_176/177_3'3'_GC**	AGTCATTTGCCTTTAGGATTTTTCTGGATTGTCCTTCCAGTTTCACCCTTCTCCTGGGTG
**MALAT1_12_176/177_3'3'_GC**	AACTGTAAACCTGTGGTGGTTTTCTGGATTGTCCTTCCAGTTTCACCCTTCTCCTGGGTG
**MALAT1_13_176/177_3'3'_GC**	CTCAACGTGAGAACTGCTCTTTTCTGGATTGTCCTTCCAGTTTCACCCTTCTCCTGGGTG
**MALAT1_14_176/177_3'3'_GC**	CTCTAACCCAGTTTGTCAATTTTCTGGATTGTCCTTCCAGTTTCACCCTTCTCCTGGGTG
**MALAT1_15_176/177_3'3'_GC**	CCAAGGATAAAAGCAGCTCCTTTCTGGATTGTCCTTCCAGTTTCACCCTTCTCCTGGGTG
**MALAT1_16_176/177_3'3'_GC**	TGAACCAAAGCTGCACTGTGTTTCTGGATTGTCCTTCCAGTTTCACCCTTCTCCTGGGTG
**MALAT1_17_176/177_3'3'_GC**	ACTGCCAACTAATTGCCAATTTTCTGGATTGTCCTTCCAGTTTCACCCTTCTCCTGGGTG
**MALAT1_18_176/177_3'3'_GC**	CCAGTGGCTCATATTTAACTTTTCTGGATTGTCCTTCCAGTTTCACCCTTCTCCTGGGTG
**MALAT1_19_176/177_3'3'_GC**	ACTTTCCTTGCCCAAATTAATTTCTGGATTGTCCTTCCAGTTTCACCCTTCTCCTGGGTG
**MALAT1_20_176/177_3'3'_GC**	CCCAATGGAGGTATGACATATTTCTGGATTGTCCTTCCAGTTTCACCCTTCTCCTGGGTG
**MALAT1_21_176/177_3'3'_GC**	CATGCAATACTGCAGATGCATTTCTGGATTGTCCTTCCAGTTTCACCCTTCTCCTGGGTG
**MALAT1_22_176/177_3'3'_GC**	AGGAGAAAGTGCCATGGTTGTTTCTGGATTGTCCTTCCAGTTTCACCCTTCTCCTGGGTG
**MALAT1_23_176/177_3'3'_GC**	TTTCTCAATCCTGAAATCCCTTTCTGGATTGTCCTTCCAGTTTCACCCTTCTCCTGGGTG
**MALAT1_24_176/177_3'3'_GC**	TGAAGTGTACTATCCCATCATTTCTGGATTGTCCTTCCAGTTTCACCCTTCTCCTGGGTG
**MALAT1_25_176/177_3'3'_GC**	TCCTGATCTGGTCCATTAAATTTCTGGATTGTCCTTCCAGTTTCACCCTTCTCCTGGGTG
**MALAT1_26_176/177_3'3'_GC**	TCTTTCCTGCCTTAAAGTTATTTCTGGATTGTCCTTCCAGTTTCACCCTTCTCCTGGGTG
**MALAT1_27_176/177_3'3'_GC**	TGTCAATTTATAGACCCCTGTTTCTGGATTGTCCTTCCAGTTTCACCCTTCTCCTGGGTG
**MALAT1_28_176/177_3'3'_GC**	AAACATTGCCTACCACTCTATTTCTGGATTGTCCTTCCAGTTTCACCCTTCTCCTGGGTG
**MALAT1_29_176/177_3'3'_GC**	CCTGAATGGCTTCATGAAGGTTTCTGGATTGTCCTTCCAGTTTCACCCTTCTCCTGGGTG
**MALAT1_30_176/177_3'3'_GC**	TGCATTTACTTGCCAACAGATTTCTGGATTGTCCTTCCAGTTTCACCCTTCTCCTGGGTG
**MALAT1_31_176/177_3'3'_GC**	GTCGTTTCACAATGCATTCTTTTCTGGATTGTCCTTCCAGTTTCACCCTTCTCCTGGGTG
**MALAT1_32_176/177_3'3'_GC**	CAACACTCAGCCTTTATCACTTTCTGGATTGTCCTTCCAGTTTCACCCTTCTCCTGGGTG
**MALAT1_33_176/177_3'3'_GC**	TTTTTTCTTACTGGGTCTGGTTTCTGGATTGTCCTTCCAGTTTCACCCTTCTCCTGGGTG
**MALAT1_34_176/177_3'3'_GC**	ACTGGAAGCTCCTTCTATAGTTTCTGGATTGTCCTTCCAGTTTCACCCTTCTCCTGGGTG
**MALAT1_35_176/177_3'3'_GC**	TCATTTTGTCCACTGGTGAATTTCTGGATTGTCCTTCCAGTTTCACCCTTCTCCTGGGTG
**MALAT1_36_176/177_3'3'_GC**	TTGTCCCATAACTGATCTGATTTCTGGATTGTCCTTCCAGTTTCACCCTTCTCCTGGGTG
**MALAT1_37_176/177_3'3'_GC**	AACACAGTTTGCTCACATGCTTTCTGGATTGTCCTTCCAGTTTCACCCTTCTCCTGGGTG
**MALAT1_38_176/177_3'3'_GC**	TGACACTTCTCTTGACCTTATTTCTGGATTGTCCTTCCAGTTTCACCCTTCTCCTGGGTG
**MALAT1_39_176/177_3'3'_GC**	CACTCCAGAAAGAGGGAGTTTTTCTGGATTGTCCTTCCAGTTTCACCCTTCTCCTGGGTG
**MALAT1_40_176/177_3'3'_GC**	AGATCAAAAGGCACGGGGTGTTTCTGGATTGTCCTTCCAGTTTCACCCTTCTCCTGGGTG
**MALAT1_41_176/177_3'3'_GC**	ACAAGGATCCAAGCTACTGGTTTCTGGATTGTCCTTCCAGTTTCACCCTTCTCCTGGGTG
**MALAT1_42_176/177_3'3'_GC**	CATCGTTACCTTGAAACCGATTTCTGGATTGTCCTTCCAGTTTCACCCTTCTCCTGGGTG
**MALAT1_43_176/177_3'3'_GC**	TAACATAGTTCAACCCACCATTTCTGGATTGTCCTTCCAGTTTCACCCTTCTCCTGGGTG
**MALAT1_44_176/177_3'3'_GC**	TTGCAGGCAAATTAATGGCCTTTCTGGATTGTCCTTCCAGTTTCACCCTTCTCCTGGGTG
**MALAT1_45_176/177_3'3'_GC**	TGTGGTTTTAATACCCTTCTTTTCTGGATTGTCCTTCCAGTTTCACCCTTCTCCTGGGTG
**MALAT1_46_176/177_3'3'_GC**	TTTCTCCACTTACTGGTTTATTTCTGGATTGTCCTTCCAGTTTCACCCTTCTCCTGGGTG
**MALAT1_47_176/177_3'3'_GC**	CTATCTTCTCCAGTCTACAATTTCTGGATTGTCCTTCCAGTTTCACCCTTCTCCTGGGTG
**MALAT1_48_176/177_3'3'_GC**	CAAGTCTGTTATGTTCACCTTTTCTGGATTGTCCTTCCAGTTTCACCCTTCTCCTGGGTG

Table S8. MALAT 1 double label probe set.

Probe	Sequence
**MALAT1_1_176/177_3'3'_GG**	GCCCTTCTATTGGTATTAATTTTCTGGATTGTCCTTCCAGTTTCACCCTTGTCCTGGGTG
**MALAT1_2_176/177_3'3'_GG**	CTCCCAATTAATCTTTCCATTTTCTGGATTGTCCTTCCAGTTTCACCCTTGTCCTGGGTG
**MALAT1_3_176/177_3'3'_GG**	TCTCCAAATTGTTTCATCCTTTTCTGGATTGTCCTTCCAGTTTCACCCTTGTCCTGGGTG
**MALAT1_4_176/177_3'3'_GG**	TTAGCTTTTTGTTTCCTAGCTTTCTGGATTGTCCTTCCAGTTTCACCCTTGTCCTGGGTG
**MALAT1_5_176/177_3'3'_GG**	ATCTTCTCAAGCTTTACCTTTTTCTGGATTGTCCTTCCAGTTTCACCCTTGTCCTGGGTG
**MALAT1_6_176/177_3'3'_GG**	ACTATATTTAAGGCCTTCCATTTCTGGATTGTCCTTCCAGTTTCACCCTTGTCCTGGGTG
**MALAT1_7_176/177_3'3'_GG**	TACTTCCGTTACGAAAGTCCTTTCTGGATTGTCCTTCCAGTTTCACCCTTGTCCTGGGTG
**MALAT1_8_176/177_3'3'_GG**	CTGGGTCAGCTGTCAATTAATTTCTGGATTGTCCTTCCAGTTTCACCCTTGTCCTGGGTG
**MALAT1_9_176/177_3'3'_GG**	TCAGTCCTAGCTTCATCAAATTTCTGGATTGTCCTTCCAGTTTCACCCTTGTCCTGGGTG
**MALAT1_10_176/177_3'3'_GG**	AACAACATATTGCCGACCTCTTTCTGGATTGTCCTTCCAGTTTCACCCTTGTCCTGGGTG
**MALAT1_11_176/177_3'3'_GG**	AGTCATTTGCCTTTAGGATTTTTCTGGATTGTCCTTCCAGTTTCACCCTTGTCCTGGGTG
**MALAT1_12_176/177_3'3'_GG**	AACTGTAAACCTGTGGTGGTTTTCTGGATTGTCCTTCCAGTTTCACCCTTGTCCTGGGTG
**MALAT1_13_176/177_3'3'_GG**	CTCAACGTGAGAACTGCTCTTTTCTGGATTGTCCTTCCAGTTTCACCCTTGTCCTGGGTG
**MALAT1_14_176/177_3'3'_GG**	CTCTAACCCAGTTTGTCAATTTTCTGGATTGTCCTTCCAGTTTCACCCTTGTCCTGGGTG
**MALAT1_15_176/177_3'3'_GG**	CCAAGGATAAAAGCAGCTCCTTTCTGGATTGTCCTTCCAGTTTCACCCTTGTCCTGGGTG
**MALAT1_16_176/177_3'3'_GG**	TGAACCAAAGCTGCACTGTGTTTCTGGATTGTCCTTCCAGTTTCACCCTTGTCCTGGGTG
**MALAT1_17_176/177_3'3'_GG**	ACTGCCAACTAATTGCCAATTTTCTGGATTGTCCTTCCAGTTTCACCCTTGTCCTGGGTG
**MALAT1_18_176/177_3'3'_GG**	CCAGTGGCTCATATTTAACTTTTCTGGATTGTCCTTCCAGTTTCACCCTTGTCCTGGGTG
**MALAT1_19_176/177_3'3'_GG**	ACTTTCCTTGCCCAAATTAATTTCTGGATTGTCCTTCCAGTTTCACCCTTGTCCTGGGTG
**MALAT1_20_176/177_3'3'_GG**	CCCAATGGAGGTATGACATATTTCTGGATTGTCCTTCCAGTTTCACCCTTGTCCTGGGTG
**MALAT1_21_176/177_3'3'_GG**	CATGCAATACTGCAGATGCATTTCTGGATTGTCCTTCCAGTTTCACCCTTGTCCTGGGTG
**MALAT1_22_176/177_3'3'_GG**	AGGAGAAAGTGCCATGGTTGTTTCTGGATTGTCCTTCCAGTTTCACCCTTGTCCTGGGTG
**MALAT1_23_176/177_3'3'_GG**	TTTCTCAATCCTGAAATCCCTTTCTGGATTGTCCTTCCAGTTTCACCCTTGTCCTGGGTG
**MALAT1_24_176/177_3'3'_GG**	TGAAGTGTACTATCCCATCATTTCTGGATTGTCCTTCCAGTTTCACCCTTGTCCTGGGTG
**MALAT1_25_176/177_3'3'_GG**	TCCTGATCTGGTCCATTAAATTTCTGGATTGTCCTTCCAGTTTCACCCTTGTCCTGGGTG
**MALAT1_26_176/177_3'3'_GG**	TCTTTCCTGCCTTAAAGTTATTTCTGGATTGTCCTTCCAGTTTCACCCTTGTCCTGGGTG
**MALAT1_27_176/177_3'3'_GG**	TGTCAATTTATAGACCCCTGTTTCTGGATTGTCCTTCCAGTTTCACCCTTGTCCTGGGTG
**MALAT1_28_176/177_3'3'_GG**	AAACATTGCCTACCACTCTATTTCTGGATTGTCCTTCCAGTTTCACCCTTGTCCTGGGTG
**MALAT1_29_176/177_3'3'_GG**	CCTGAATGGCTTCATGAAGGTTTCTGGATTGTCCTTCCAGTTTCACCCTTGTCCTGGGTG
**MALAT1_30_176/177_3'3'_GG**	TGCATTTACTTGCCAACAGATTTCTGGATTGTCCTTCCAGTTTCACCCTTGTCCTGGGTG
**MALAT1_31_176/177_3'3'_GG**	GTCGTTTCACAATGCATTCTTTTCTGGATTGTCCTTCCAGTTTCACCCTTGTCCTGGGTG
**MALAT1_32_176/177_3'3'_GG**	CAACACTCAGCCTTTATCACTTTCTGGATTGTCCTTCCAGTTTCACCCTTGTCCTGGGTG
**MALAT1_33_176/177_3'3'_GG**	TTTTTTCTTACTGGGTCTGGTTTCTGGATTGTCCTTCCAGTTTCACCCTTGTCCTGGGTG
**MALAT1_34_176/177_3'3'_GG**	ACTGGAAGCTCCTTCTATAGTTTCTGGATTGTCCTTCCAGTTTCACCCTTGTCCTGGGTG
**MALAT1_35_176/177_3'3'_GG**	TCATTTTGTCCACTGGTGAATTTCTGGATTGTCCTTCCAGTTTCACCCTTGTCCTGGGTG
**MALAT1_36_176/177_3'3'_GG**	TTGTCCCATAACTGATCTGATTTCTGGATTGTCCTTCCAGTTTCACCCTTGTCCTGGGTG
**MALAT1_37_176/177_3'3'_GG**	AACACAGTTTGCTCACATGCTTTCTGGATTGTCCTTCCAGTTTCACCCTTGTCCTGGGTG
**MALAT1_38_176/177_3'3'_GG**	TGACACTTCTCTTGACCTTATTTCTGGATTGTCCTTCCAGTTTCACCCTTGTCCTGGGTG
**MALAT1_39_176/177_3'3'_GG**	CACTCCAGAAAGAGGGAGTTTTTCTGGATTGTCCTTCCAGTTTCACCCTTGTCCTGGGTG
**MALAT1_40_176/177_3'3'_GG**	AGATCAAAAGGCACGGGGTGTTTCTGGATTGTCCTTCCAGTTTCACCCTTGTCCTGGGTG
**MALAT1_41_176/177_3'3'_GG**	ACAAGGATCCAAGCTACTGGTTTCTGGATTGTCCTTCCAGTTTCACCCTTGTCCTGGGTG
**MALAT1_42_176/177_3'3'_GG**	CATCGTTACCTTGAAACCGATTTCTGGATTGTCCTTCCAGTTTCACCCTTGTCCTGGGTG
**MALAT1_43_176/177_3'3'_GG**	TAACATAGTTCAACCCACCATTTCTGGATTGTCCTTCCAGTTTCACCCTTGTCCTGGGTG
**MALAT1_44_176/177_3'3'_GG**	TTGCAGGCAAATTAATGGCCTTTCTGGATTGTCCTTCCAGTTTCACCCTTGTCCTGGGTG
**MALAT1_45_176/177_3'3'_GG**	TGTGGTTTTAATACCCTTCTTTTCTGGATTGTCCTTCCAGTTTCACCCTTGTCCTGGGTG
**MALAT1_46_176/177_3'3'_GG**	TTTCTCCACTTACTGGTTTATTTCTGGATTGTCCTTCCAGTTTCACCCTTGTCCTGGGTG
**MALAT1_47_176/177_3'3'_GG**	CTATCTTCTCCAGTCTACAATTTCTGGATTGTCCTTCCAGTTTCACCCTTGTCCTGGGTG
**MALAT1_48_176/177_3'3'_GG**	CAAGTCTGTTATGTTCACCTTTTCTGGATTGTCCTTCCAGTTTCACCCTTGTCCTGGGTG

11. **Supplementary Figures**

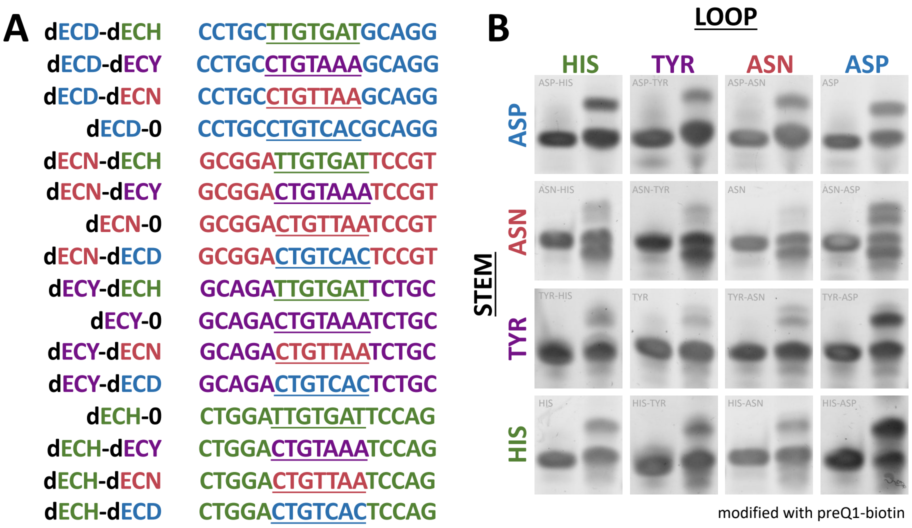

Figure S1. TGT labeling of DNA analog chimeras of queuine cognate tRNA anticodon loops. **(A)** Hairpin Sequences. **(B)** Urea PAGE gel shift analysis. For gel data, unmodified oligos are in the left lane and reaction products are in the right lane. Modification is determined by an upward gel shift of the oligo after insertion of preQ1-biotin.

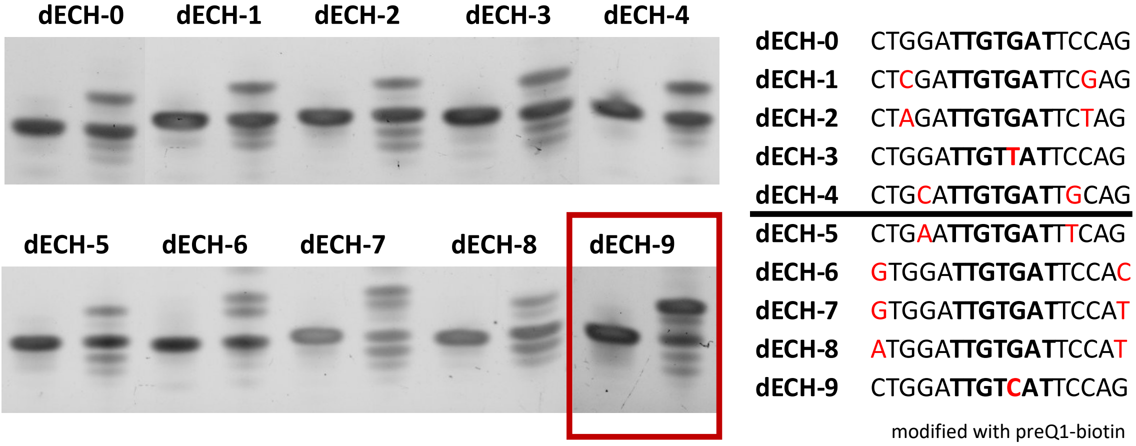

Figure S2. TGT modification of histidine hairpin mutants dECH1-9 with preQ1-biotin. For gel data, unmodified oligos are in the left lane and reaction products are in the right lane. Modification is determined by an upward gel shift of the oligo after insertion of preQ1-biotin.

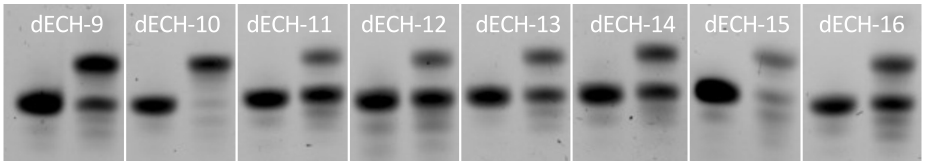

dECH-9	CTGGATTGTCATTCCAG	dECH-13	CTGGATTGTCTTTCCAG
dECH-10	CTGGATTGTCCTTCCAG	dECH-14	CTGGATTGTCGTTCCAG
dECH-11	CTGGATTGTTTTTCCAG	dECH-15	CTGGATTGTTGTTCCAG
dECH-12	CTGGATTGTTATTCCAG	dECH-16	CTGGATTGTTCTTCCAG

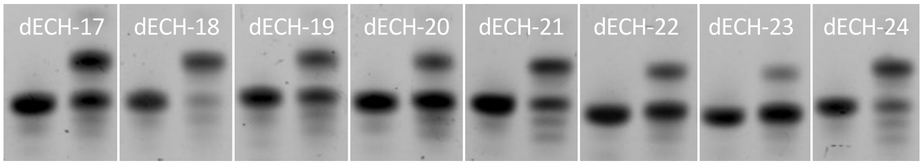

dECH-17	CTGGACTGTCACTCCAG	dECH-21	CTGGACTGTCTCTCCAG
dECH-18	CTGGACTGTCCCTCCAG	dECH-22	CTGGACTGTCGCTCCAG
dECH-19	CTGGACTGTTTCTCCAG	dECH-23	CTGGACTGTTGCTCCAG
dECH-20	CTGGACTGTTACTCCAG	dECH-24	CTGGACTGTTCCTCCAG

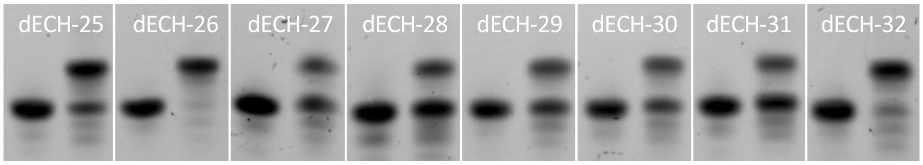

dECH-25	CTGGATTGTCACTCCAG	dECH-29	CTGGATTGTCTCTCCAG
dECH-26	CTGGATTGTCCCTCCAG	dECH-30	CTGGATTGTCGCTCCAG
dECH-27	CTGGATTGTTTCTCCAG	dECH-31	CTGGATTGTTGCTCCAG
dECH-28	CTGGATTGTTACTCCAG	dECH-32	CTGGATTGTTCCTCCAG

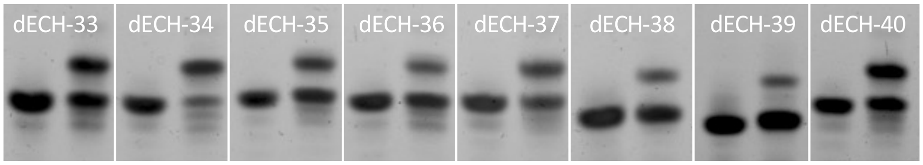

dECH-33	CTGGACTGTCATTCCAG	dECH-37	CTGGACTGTCTTTCCAG
dECH-34	CTGGACTGTCCTTCCAG	dECH-38	CTGGACTGTCGTTCCAG
dECH-35	CTGGACTGTTTTTCCAG	dECH-39	CTGGACTGTTGTTCCAG
dECH-36	CTGGACTGTTATTCCAG	dECH-40	CTGGACTGTTCTTCCAG

Figure S3. TGT modification of histidine hairpin loop mutants dECH9-40. Mutations are indicated in red. For gel data, unmodified oligos are in the left lane and reaction products are in the right lane. Modification is determined by an upward gel shift of the oligo after insertion of preQ1-biotin. Highlighted sequences are labeled with greater than 85% efficiency as determined by densitometry. The minimal loop sequence requirement is YTGTCCY or YTGTYCC where Y is a smaller pyrimidine nucleobase, C or T.

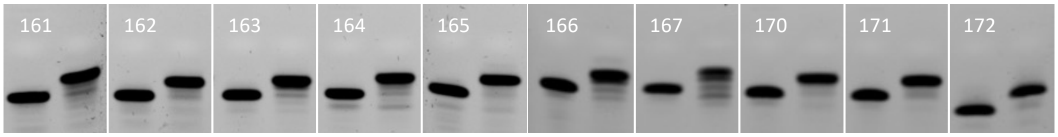

161_ala1_dECH-10	cttgcTTGTCCTgcaag	166_arg4_dECH-10	acgacTTGTCCTgtcgt
162_ala2_dECH-10	cctgcTTGTCCTgcagg	167_asn1_dECH-10	gcggaTTGTCCTtccgt
163_arg1_dECH-10	agcccTTGTCCTgggct	170_gln1_dECH-10	ccggaTTGTCCTtccgg
164_arg2_dECH-10	ctcggTTGTCCTccgag	171_gln2_dECH-10	ccggtTTGTCCTaccgg
165_arg3_dECH-10	ctgccTTGTCCTggcag	172_glu1_dECH-10	ccgccTTGTCCTggcgg

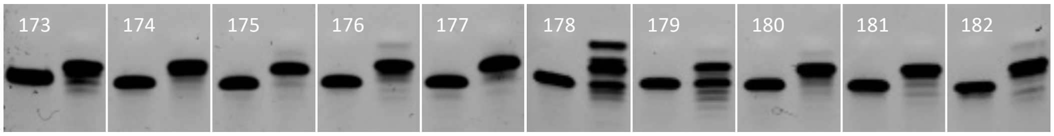

173_gly1_dECH-10	agagcTTGTCCTgctct	178_leu1_dECH-10	gttgaTTGTCCTtcaac
174_gly2_dECH-10	tcagcTTGTCCTgctga	179_leu2_dECH-10	ctagcTTGTCCTgttag
175_gly3_dECH-10	cgaccTTGTCCTggtcg	180_leu3_dECH-10	ctaccTTGTCCTggtag
176_his1_dECH-10	ctggaTTGTCCTtccag	181_leu4_dECH-10	ccagaTTGTCCTtctgg
177_ile1_dECH-10	cacccTTGTCCTgggtg	182_leu5_dECH-10	agggaTTGTCCTtccct

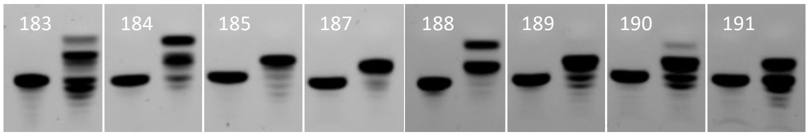

183_lys1_dECH-10	gttgaTTGTCCTtcaat	188_phe1_dECH-10	ggggaTTGTCCTtcccc
184_met1_dECH-10	ggcgaTTGTCCTtcgct	189_pro1_dECH-10	actggTTGTCCTccagt
185_met2_dECH-10	catcaTTGTCCTtgatg	190_pro2_dECH-10	ccgtcTTGTCCTgtcgg
187_met4_dECH-10	tcgggTTGTCCTcccga	191_pro3_dECH-10	cttcgTTGTCCTcgagg

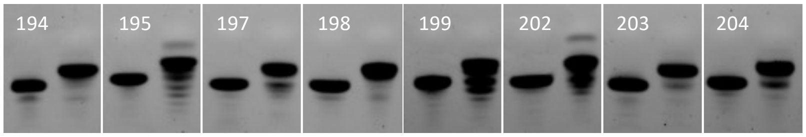

194_ser3_dECH-10	ctcccTTGTCCTgggag	199_thr4_dECH-10	actgaTTGTCCTtcagt
195_ser4_dECH-10	cacgcTTGTCCTgtgtg	202_tyr1_dECH-10	gcagaTTGTCCTtctgc
197_thr2_dECH-10	tcgcaTTGTCCTtgcga	203_val1_dECH-10	ccaccTTGTCCTggtgg
198_thr3_dECH-10	cgcatTTGTCCTatgcg	204_val2_dECH-10	cctccTTGTCCTggagg

Figure S4. TGT modification of anticodon stem/dECH-10 loop chimeras. Anticodon stems of all tRNAs were appended with the dECH-10 loop to determine the importance of stem vs loop sequence for TGT labeling. For gel data, unmodified oligos are in the left lane and reaction products are in the right lane. Modification is determined by an upward gel shift of the oligo after insertion of preQ1-biotin. Redundant hairpins where two or more tRNA anticodon arms had identical stem sequences were omitted from this figure. See Table S3 for a complete list of anticodon stem/dECH-10 loop chimeras including redundant hairpins. Highlighted sequences are labeled with greater than 85% efficiency as determined by densitometry.

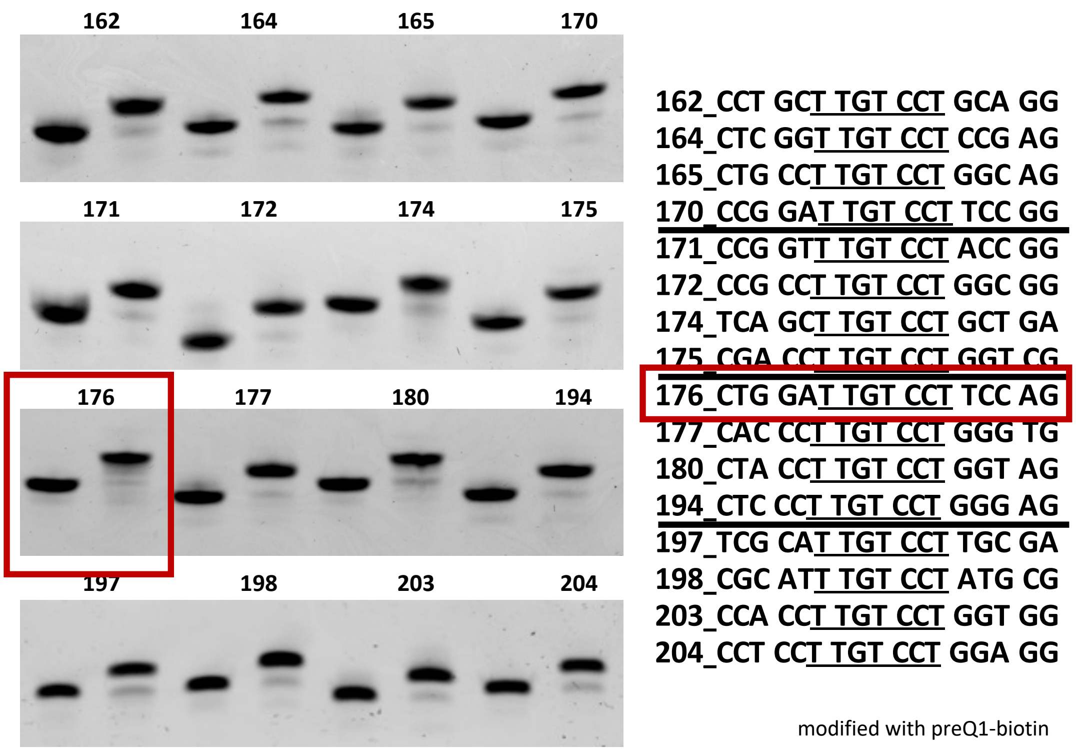

Figure S5. TGT modification of 16 distinct hairpin sequences. For gel data, unmodified oligos are in the left lane and reaction products are in the right lane. Modification is determined by an upward gel shift of the oligo after insertion of preQ1-biotin.

time (hr)	yield (%)
0	0%
0.5	45%
1	62%
2	82%
2.5	87%
3	92%
3.5	89%
4	92%
5	90%
6	95%

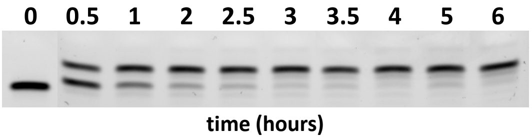

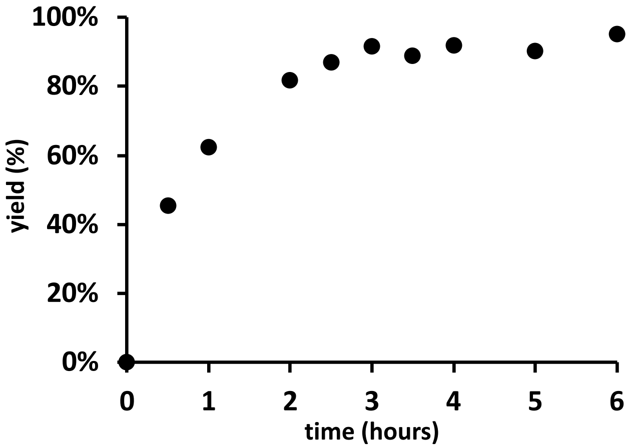

Figure S6. Time course of the modification of dECH-10. TGT insertion of preQ1-biotin into dECH-10 is monitored over time and reaches a maximum at 3-4 hours. Modification is determined by an upward gel shift of the oligo after insertion of preQ1-biotin and quantified using densitometry.

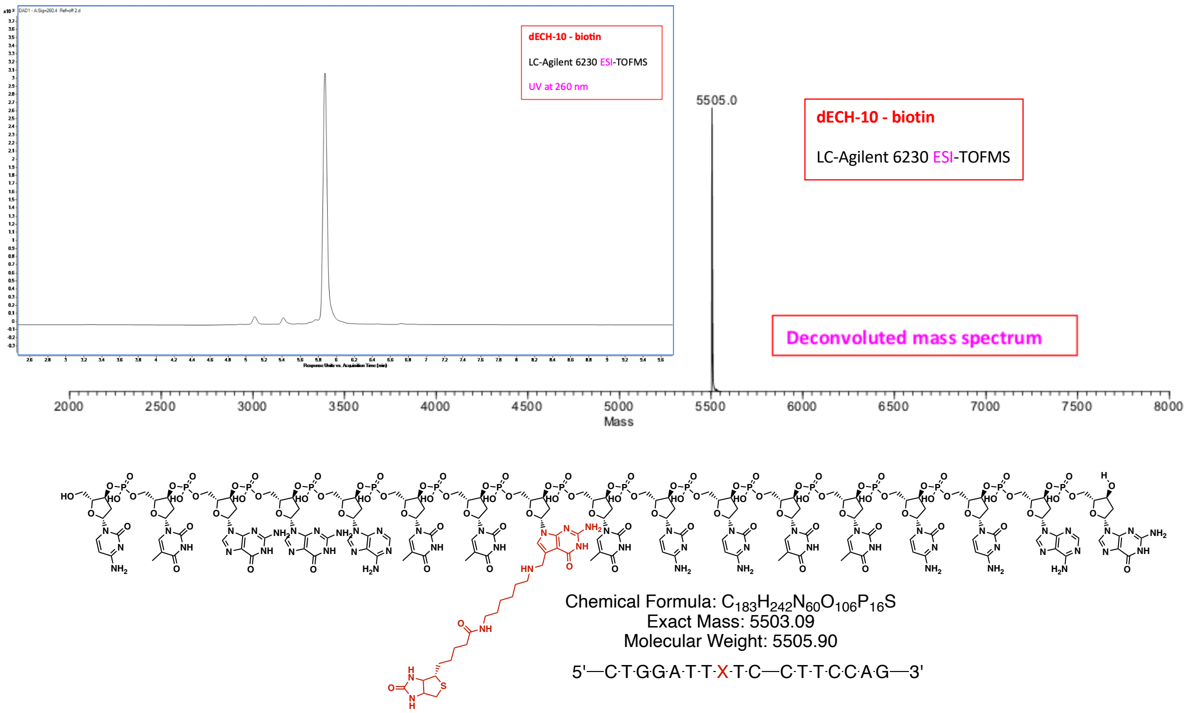

Figure S7. LCMS verification of dECH-10 modification with preQ1-biotin by *E. coli* TGT.

dECH-10 dECH-10 ΔC

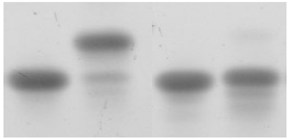

modified with PreQ1-biotin

dECH-10 CTGGA**TTG̲TCCT**TCCAG
dECH-10 ΔC CTGGA**TTC̲TCCT**TCCAG

Figure S8. Confirmation of specific guanine exchange. Mutation of the key G nucleobase to C results in no observable product. For gel data, unmodified oligos are in the left lane and reaction products are in the right lane. Modification is determined by an upward gel shift of the oligo after insertion of preQ1-biotin.

dECH-10 CTGGATTXTCCTTCCAG

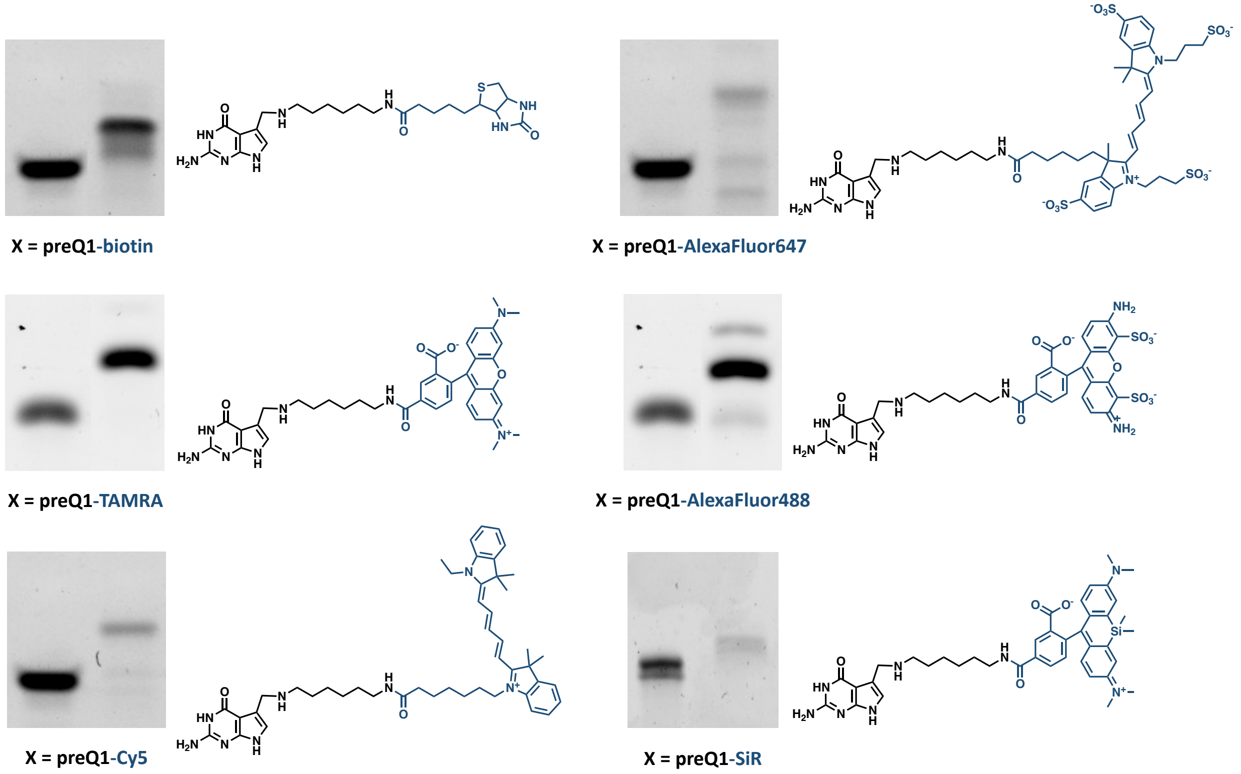

Figure S9. Substrate scope of DNA-TAG. For gel data, unmodified oligos are in the left lane and reaction products are in the right lane. Modification is determined by an upward gel shift of the oligo after insertion of of preQ1 probes. Cy5, AlexaFluor647, and Silicon Rhodamine (SiR) dyes are quenched by gel red making the product band faint. See section 12 for confirmation of labeling by MS.

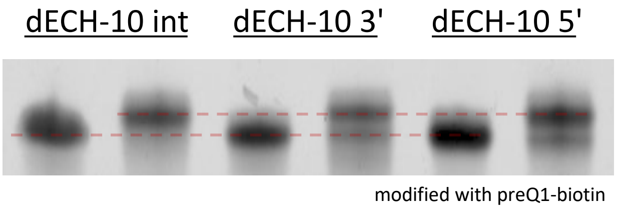

dECH-10 int ACCATCATTTTCATATCCTCCACTGGA**TTGTCCT**TCCAGACCACCATCATTTGCAATGA

dECH-10 3' ACCATCATTTTCATATCCTCCAACCACCATCATTTGCAATGACTGGA**TTGTCCT**TCCAG

dECH-10 5' CTGGA**TTGTCCT**TCCAGACCATCATTTTCATATCCTCCAACCACCATCATTTGCAATGA

Figure S10. The dECH-10 hairpin is labeled when inserted into the center, or appended to either end, of a DNA substrate. For gel data, unmodified oligos are in the left lane and reaction products are in the right lane. Modification is determined by an upward gel shift of the oligo after insertion of preQ1-biotin.

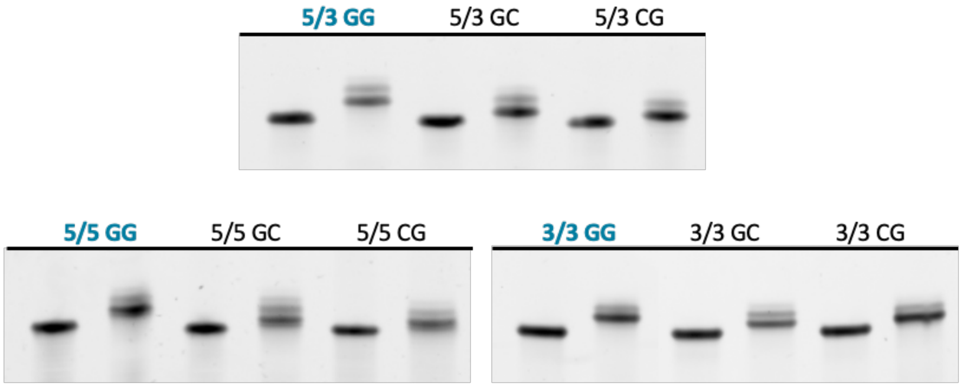

oligonucleotide	sequence	expected m/z	deconvoluted m/z
**5/3_176/177_GG**	**ctggaTTgTCCTtccagCATCATTTTCATATCCTCCAcacccTTgTCCTgggtg**	**17098.02**	**17099**
5/3_176/177_GC	ctggaTTgTCCTtccagCATCATTTTCATATCCTCCAcacccTTcTCCTgggtg	16704.81	16705
5/3_176/177_CG	ctggaTTcTCCTtccagCATCATTTTCATATCCTCCAcacccTTgTCCTgggtg	16704.81	16705
**5/5_176/177_GG**	**ctggaTTgTCCTtccagTTTcacccTTgTCCTgggtgCATCATTTTCATATCCTCCA**	**18010.62**	**18012**
5/5_176/177_GC	ctggaTTgTCCTtccagTTTcacccTTcTCCTgggtgCATCATTTTCATATCCTCCA	17617.41	17617
5/5_176/177_CG	ctggaTTcTCCTtccagTTTcacccTTgTCCTgggtgCATCATTTTCATATCCTCCA	17617.41	17617
**3/3_176/177_GG**	**CATCATTTTCATATCCTCCActggaTTgTCCTtccagTTTcacccTTgTCCTgggtg**	**18010.62**	**18011**
3/3_176/177_GC	CATCATTTTCATATCCTCCActggaTTgTCCTtccagTTTcacccTTcTCCTgggtg	17617.41	17617
3/3_176/177_CG	CATCATTTTCATATCCTCCActggaTTcTCCTtccagTTTcacccTTgTCCTgggtg	17617.41	17617

Figure S11. Single or double labeling of DNA substrates bearing orthogonal hairpins with preQ1-biotin. Single label constructs have a G→C mutation in one of the two recognition element hairpins. Modification is determined by an upward gel shift of the oligo after insertion of preQ1-biotin. For gel data, unmodified oligos are in the left lane and reaction products are in the right lane. Dual labeling was successful in tandem at either end, and when separated at both ends. Highlighted oligos indicate GG substrates.

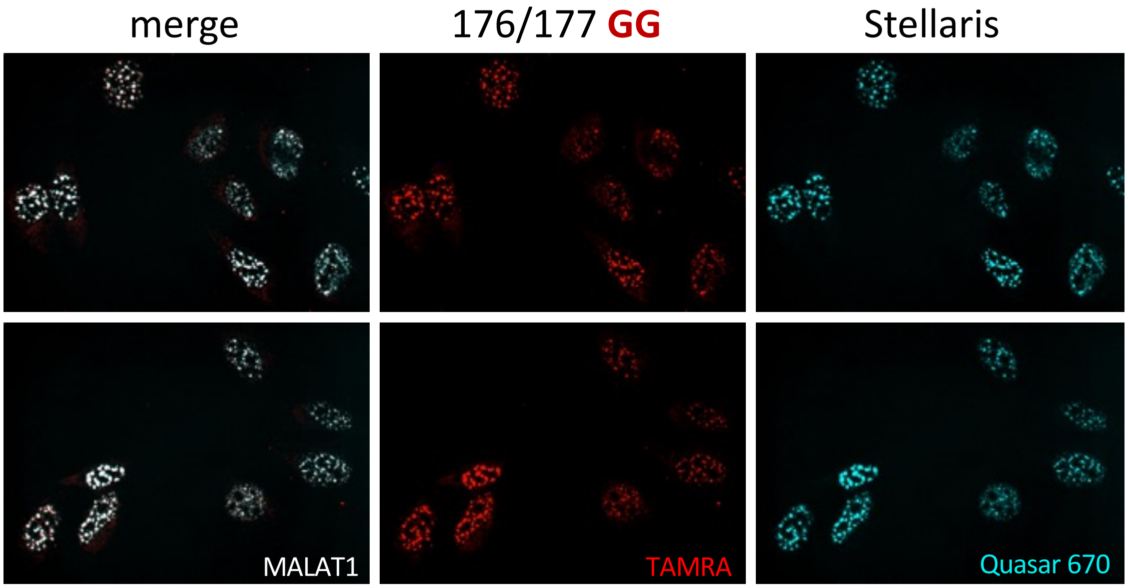

Figure S12. RNA FISH detection of MALAT1 with doubly labeled DNA-TAG generated probe set and Stellaris probe set. The DNA-TAG probe set reliably reports on MALAT1 as seen by colocalization with the commercially available probe set from Stellaris.

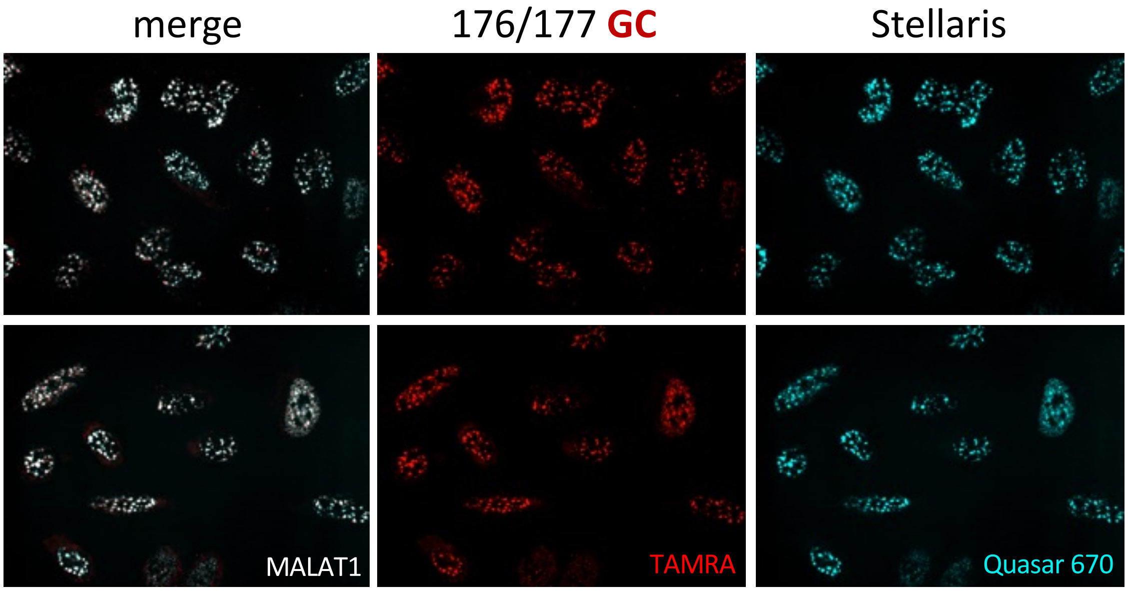

Figure S13. RNA FISH detection of MALAT1 with singly labeled DNA-TAG generated probe set and Stellaris probe set. The DNA-TAG probe set reliably reports on MALAT1 as seen by colocalization with the commercially available probe set from Stellaris.

12. MS of PreQ1 Probes

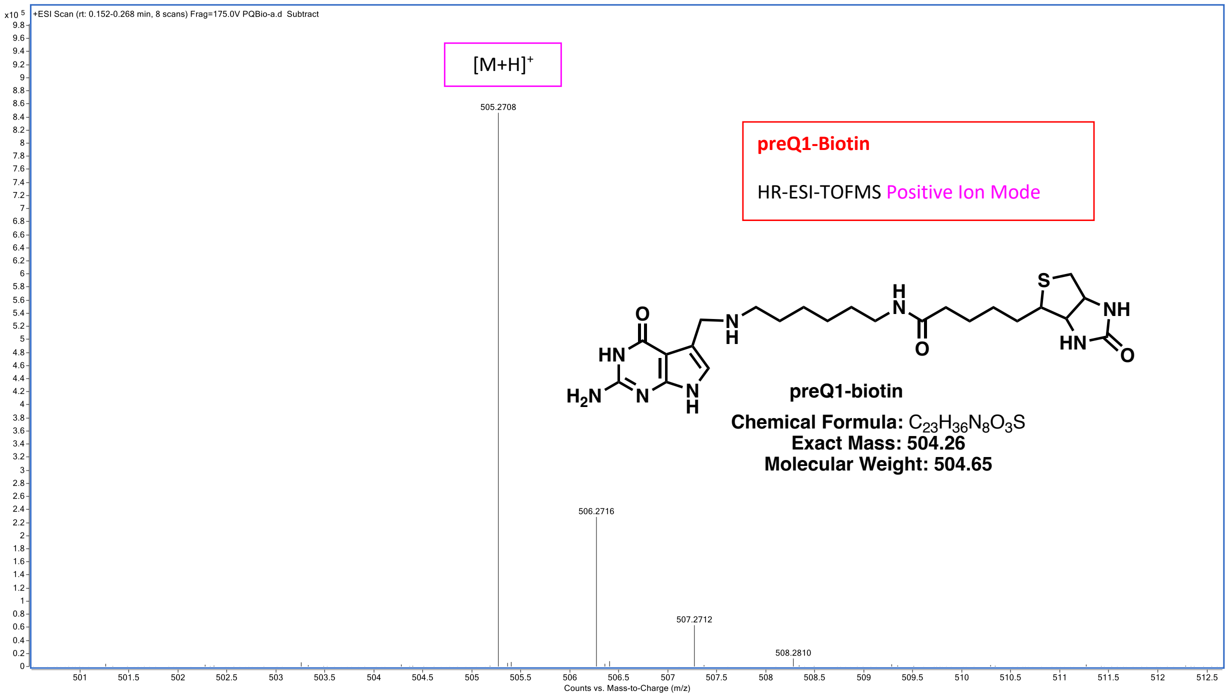

Mass Measured	Theo. Mass	Delta (ppm)	Composition
505.2708	505.2704	0.8	$[C_{23}H_{37}N_8O_3S]^+$

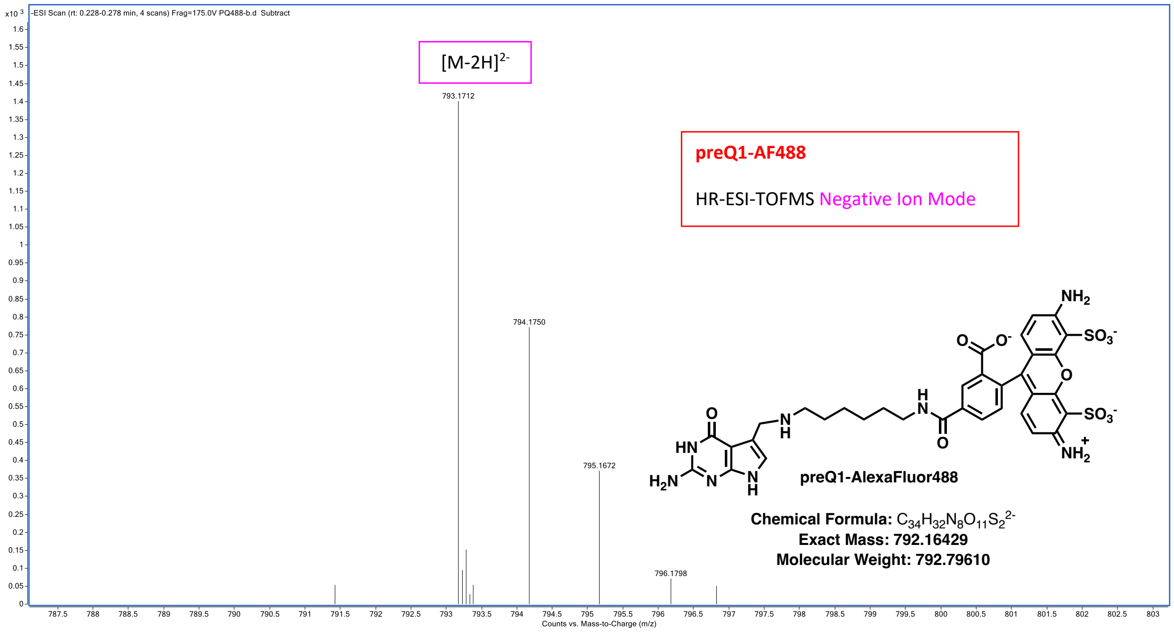

Mass Measured	Theo. Mass	Delta (ppm)	Composition
793.1712	793.1716	-0.5	$[C_{34}H_{32}N_8O_{11}S_2]^{2-}$

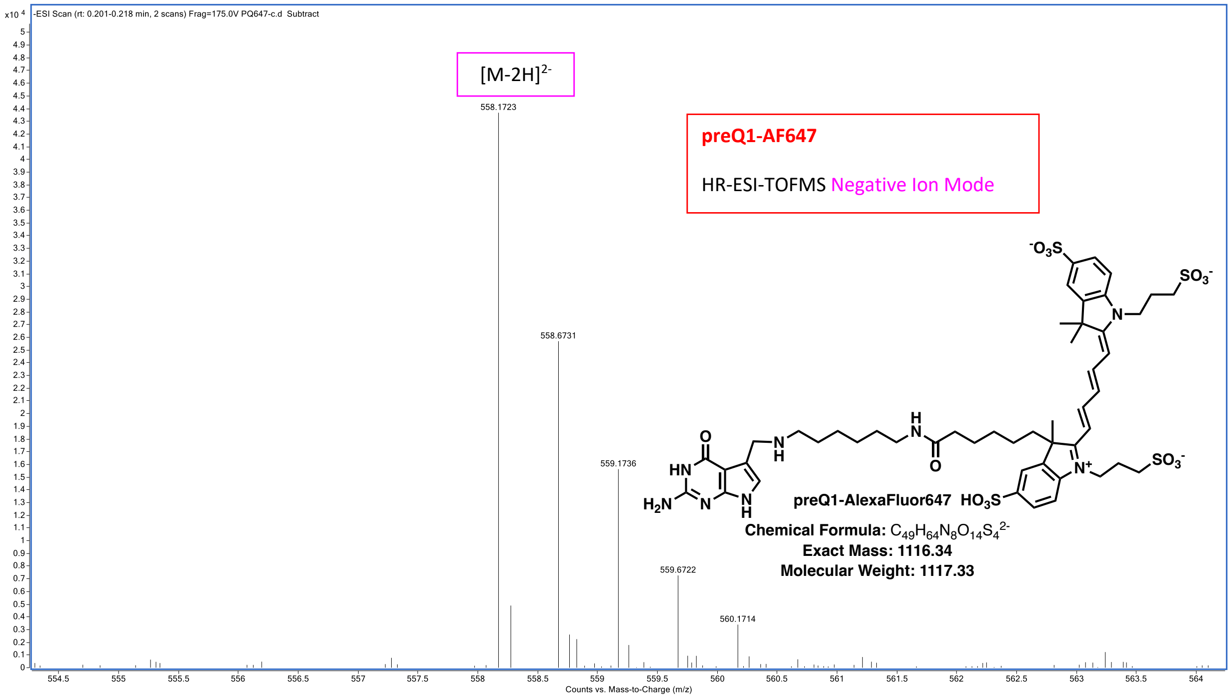

Mass Measured	Theo. Mass	Delta (ppm)	Composition
558.1723	558.1718	-0.3	$[C_{49} H_{64} N_8 O_{14} S_4]^{2-}$

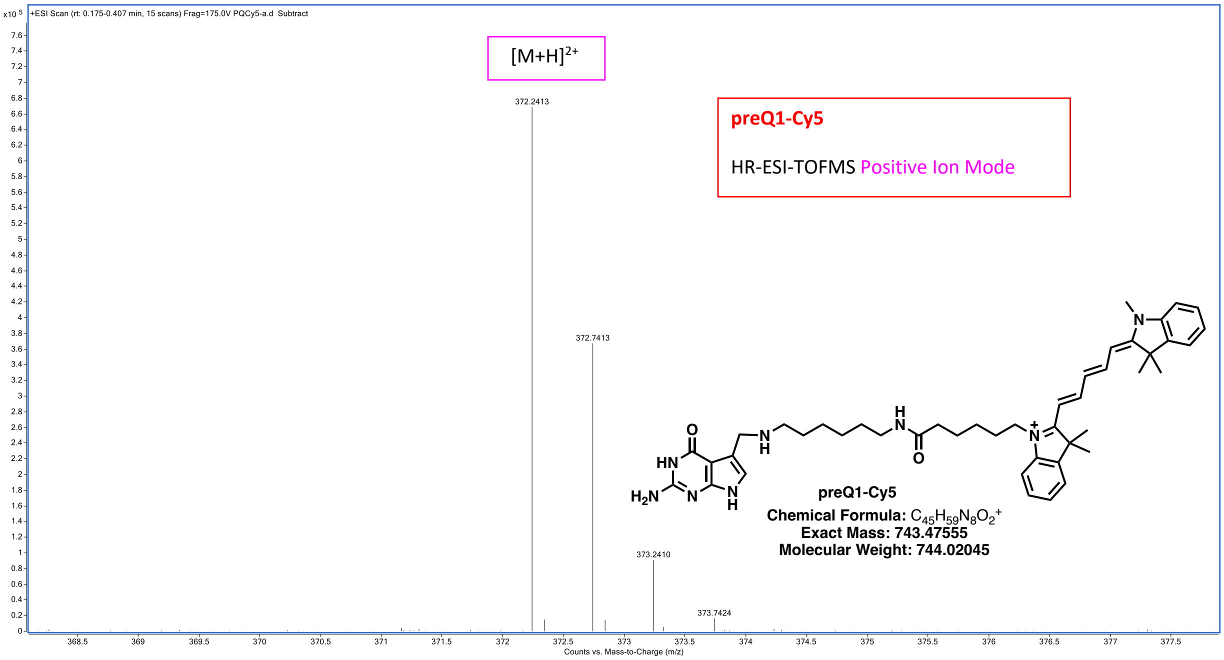

Mass Measured	Theo. Mass	Delta (ppm)	Composition
372.2413	372.2414	-0.3	$[C_{45} H_{60} N_8 O_2]^{2+}$

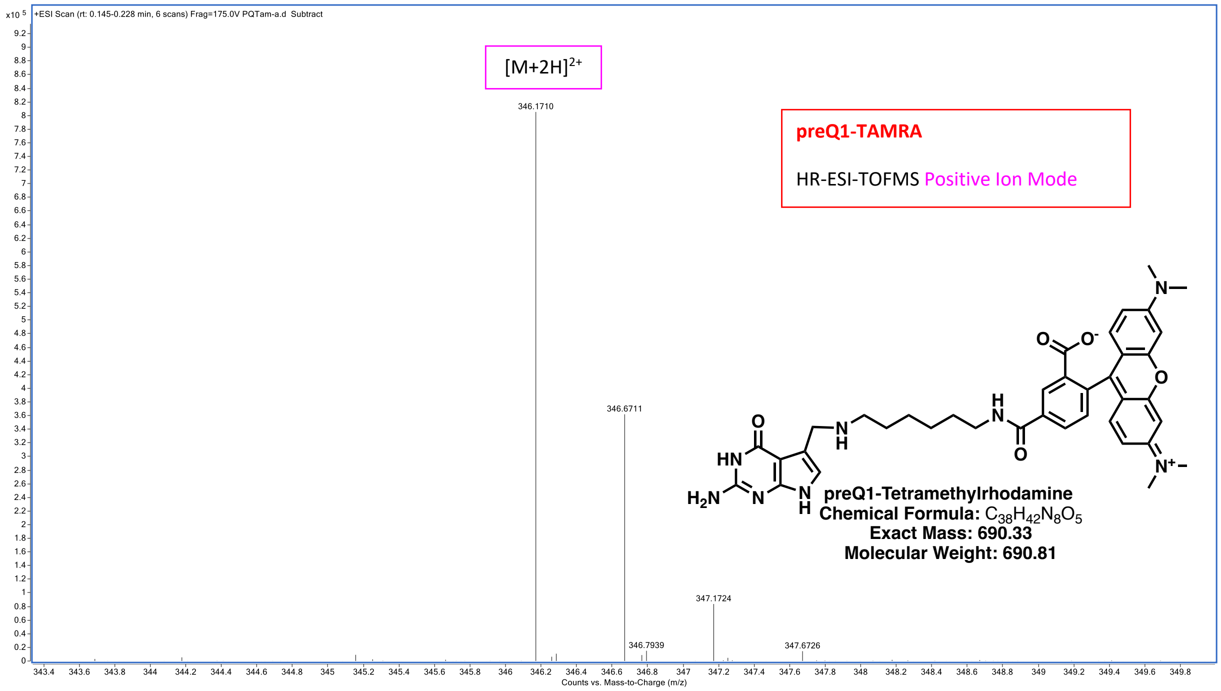

Mass Measured	Theo. Mass	Delta (ppm)	Composition
346.1710	346.1712	-0.6	$[C_{38}H_{44}N_8O_5]^{2+}$

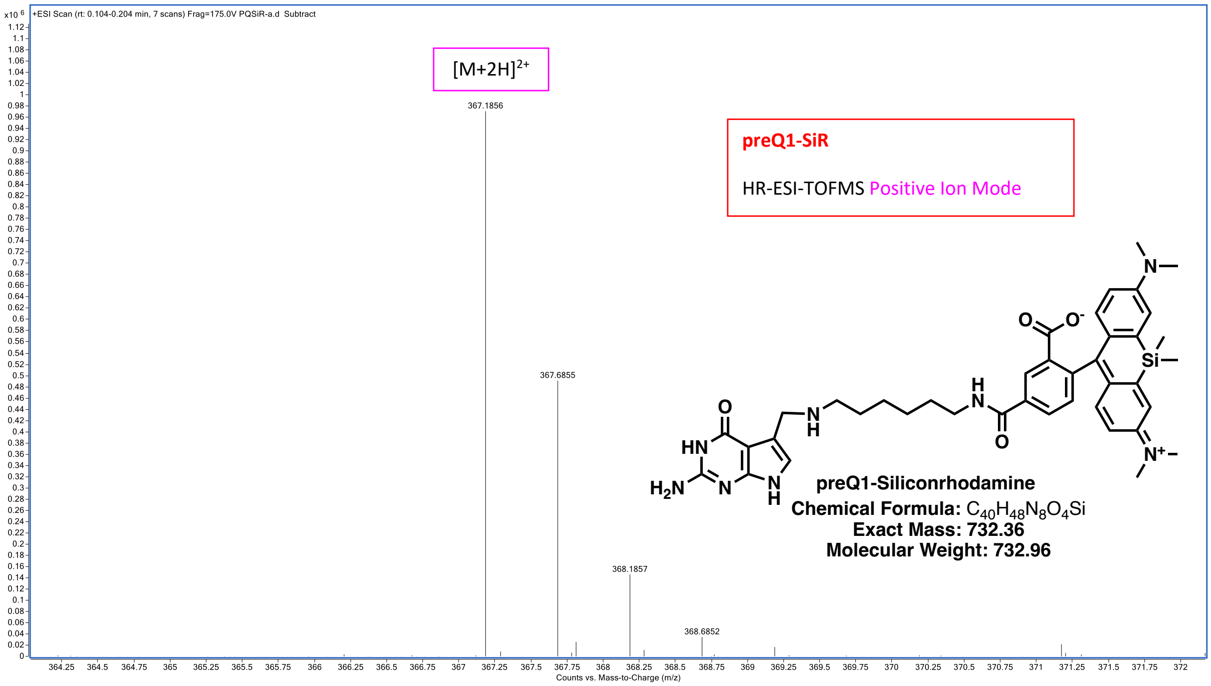

Mass Measured	Theo. Mass	Delta (ppm)	Composition
367.1856	367.1857	-0.3	$[C_{40}H_{50}N_8O_4Si]^{2+}$

13. MS of dECH-10 and modified oligos

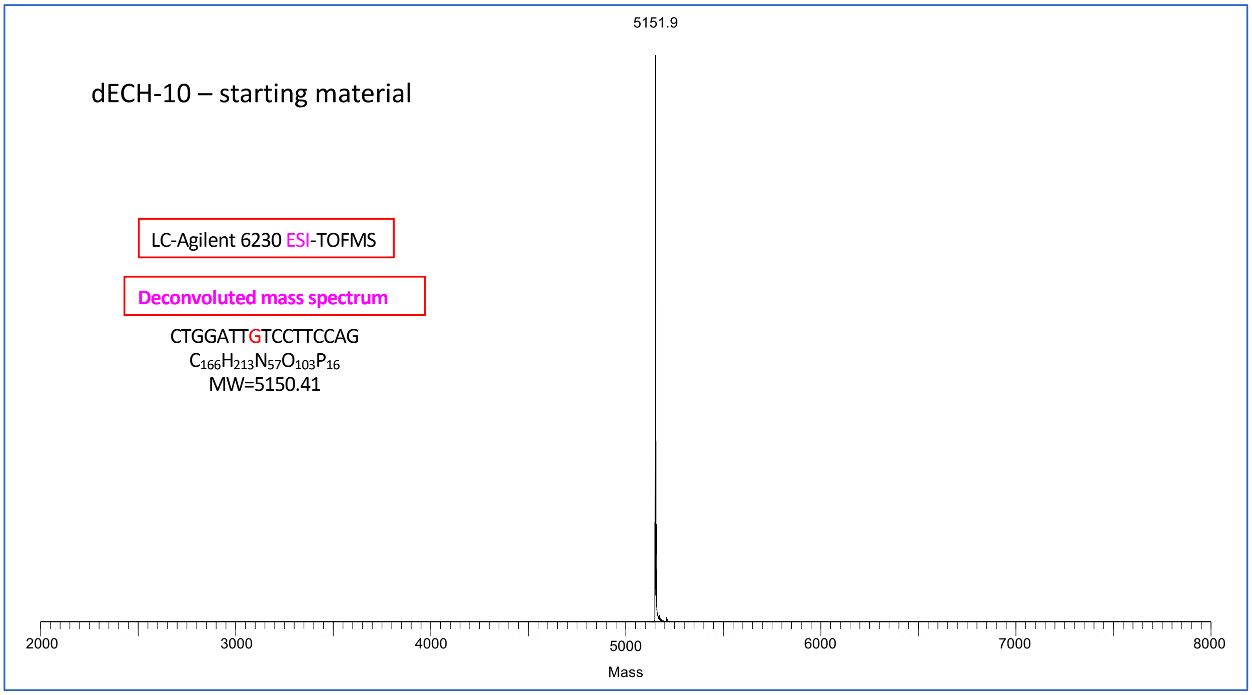

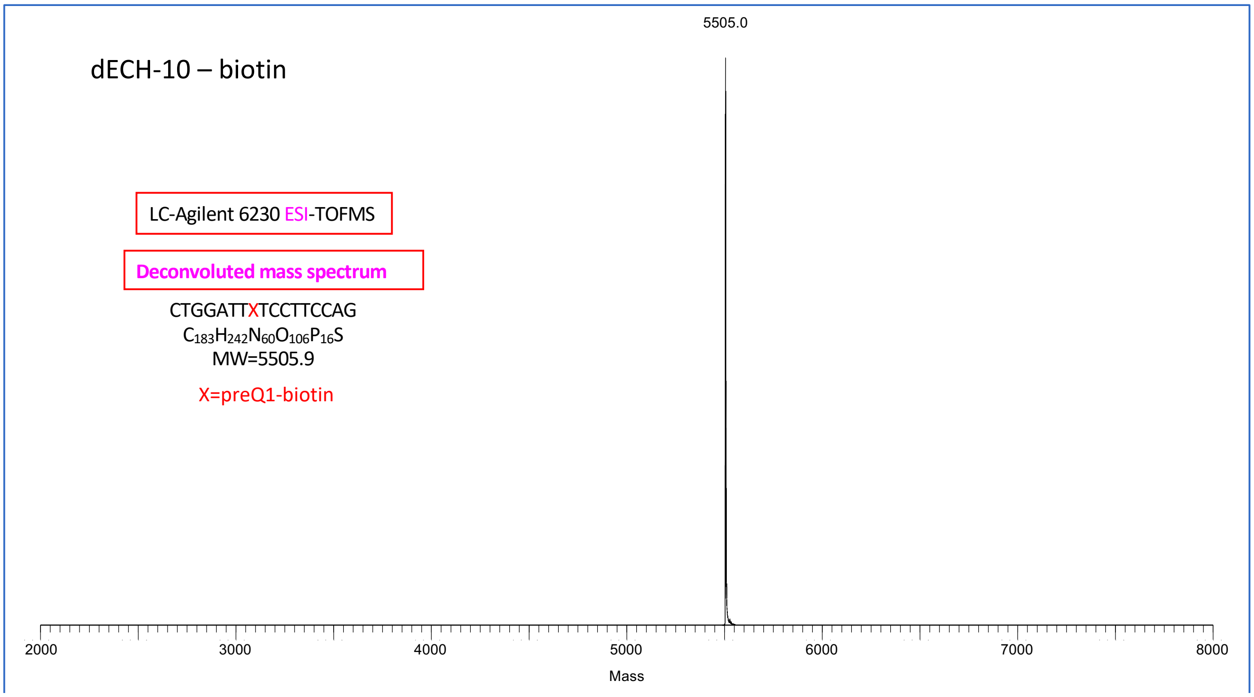

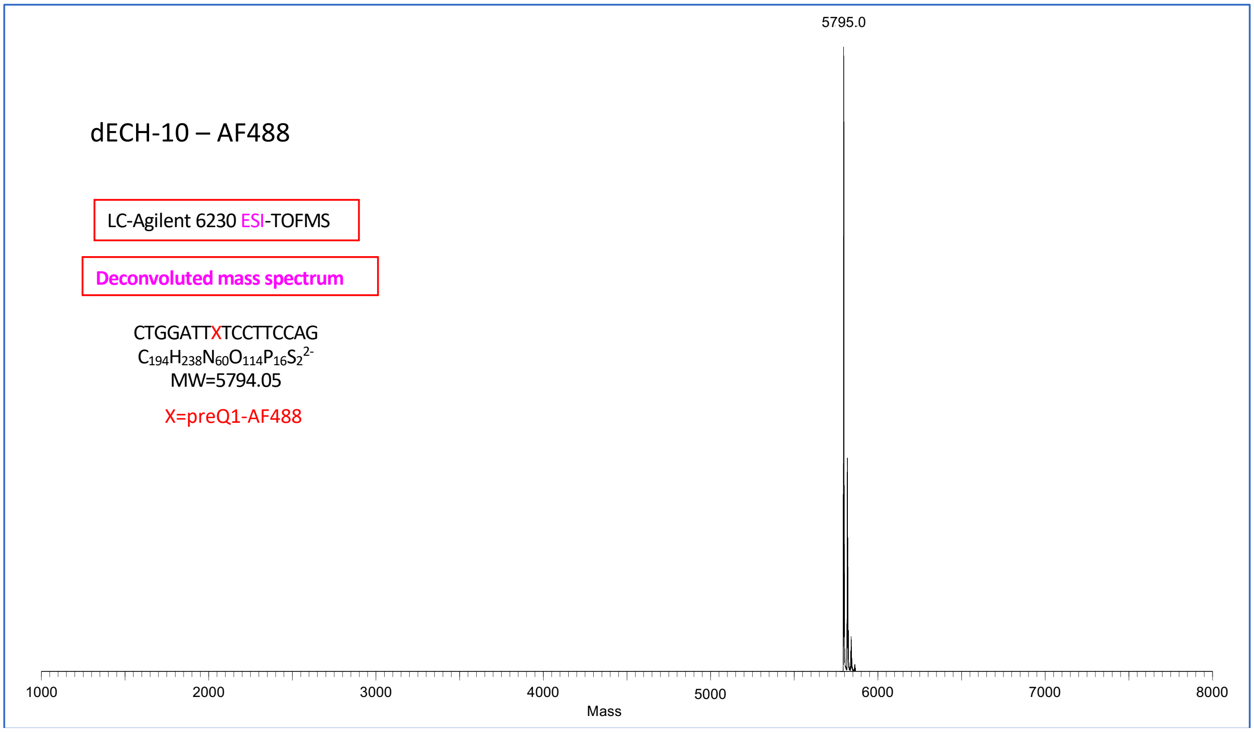
5795.0
dECH-10 – AF488
LC-Agilent 6230 ESI-TOFMS
Deconvoluted mass spectrum
CTGGATTXTCCTTCCAG
$C_{194}H_{238}N_{60}O_{114}P_{16}S_2^{2-}$
MW=5794.05
X=preQ1-AF488
1000
2000
3000
4000
5000
6000
7000
8000
Mass

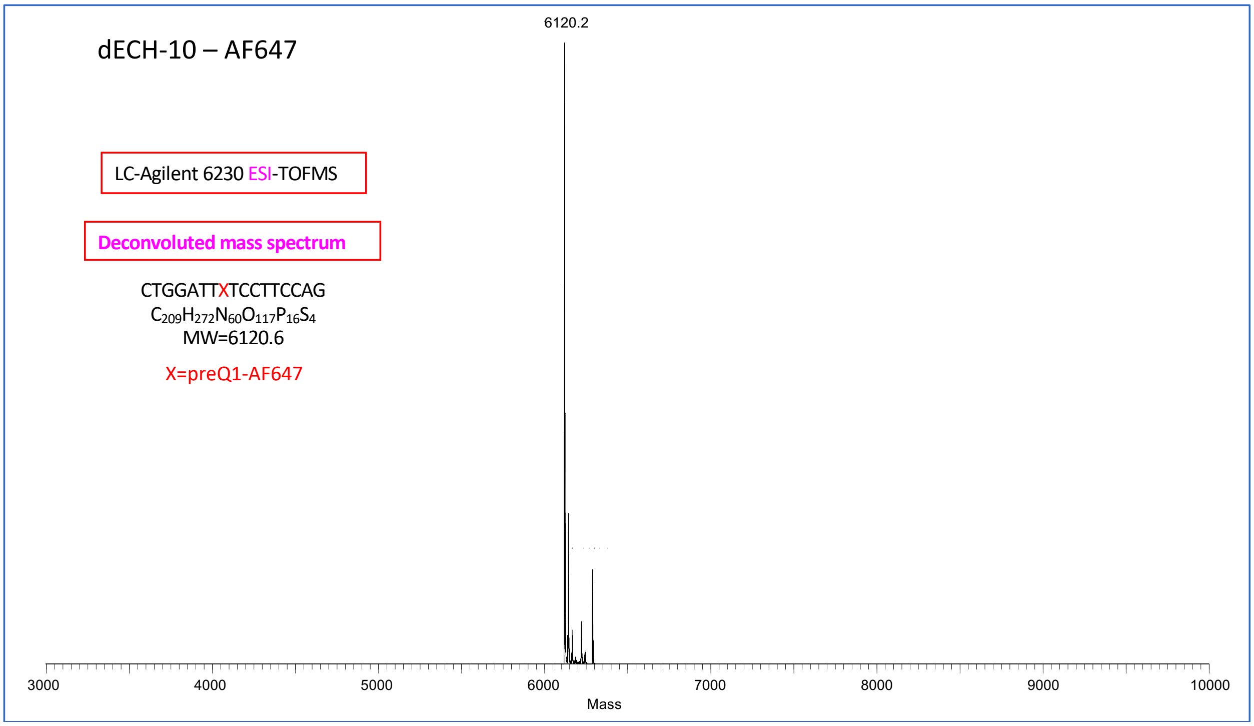
6120.2
dECH-10 – AF647
LC-Agilent 6230 ESI-TOFMS
Deconvoluted mass spectrum
CTGGATTXTCCTTCCAG
$C_{209}H_{272}N_{60}O_{117}P_{16}S_4$
MW=6120.6
X=preQ1-AF647
3000
4000
5000
6000
7000
8000
9000
10000
Mass

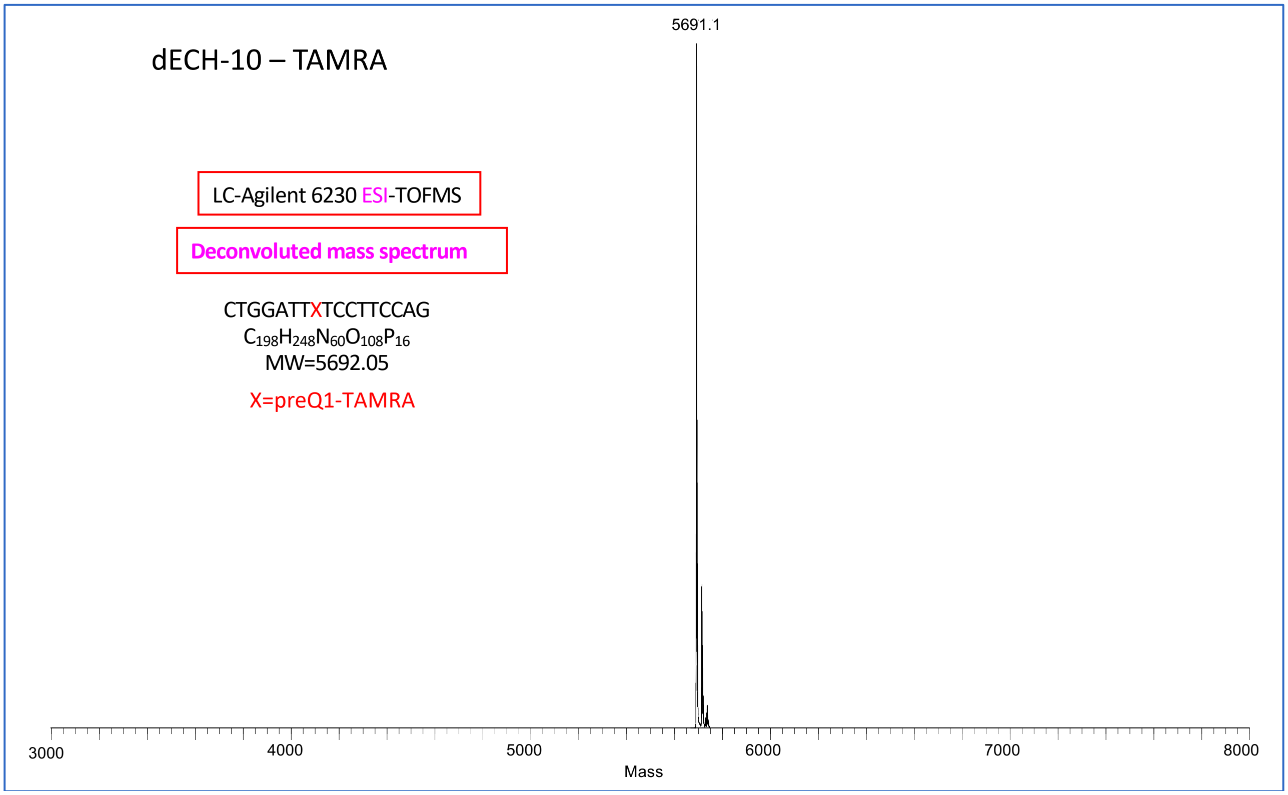
dECH-10 – TAMRA
5691.1
LC-Agilent 6230 ESI-TOFMS
Deconvoluted mass spectrum
CTGGATTXTCCTTCCAG
$C_{198}H_{248}N_{60}O_{108}P_{16}$
MW=5692.05
X=preQ1-TAMRA
3000
4000
5000
6000
7000
8000
Mass

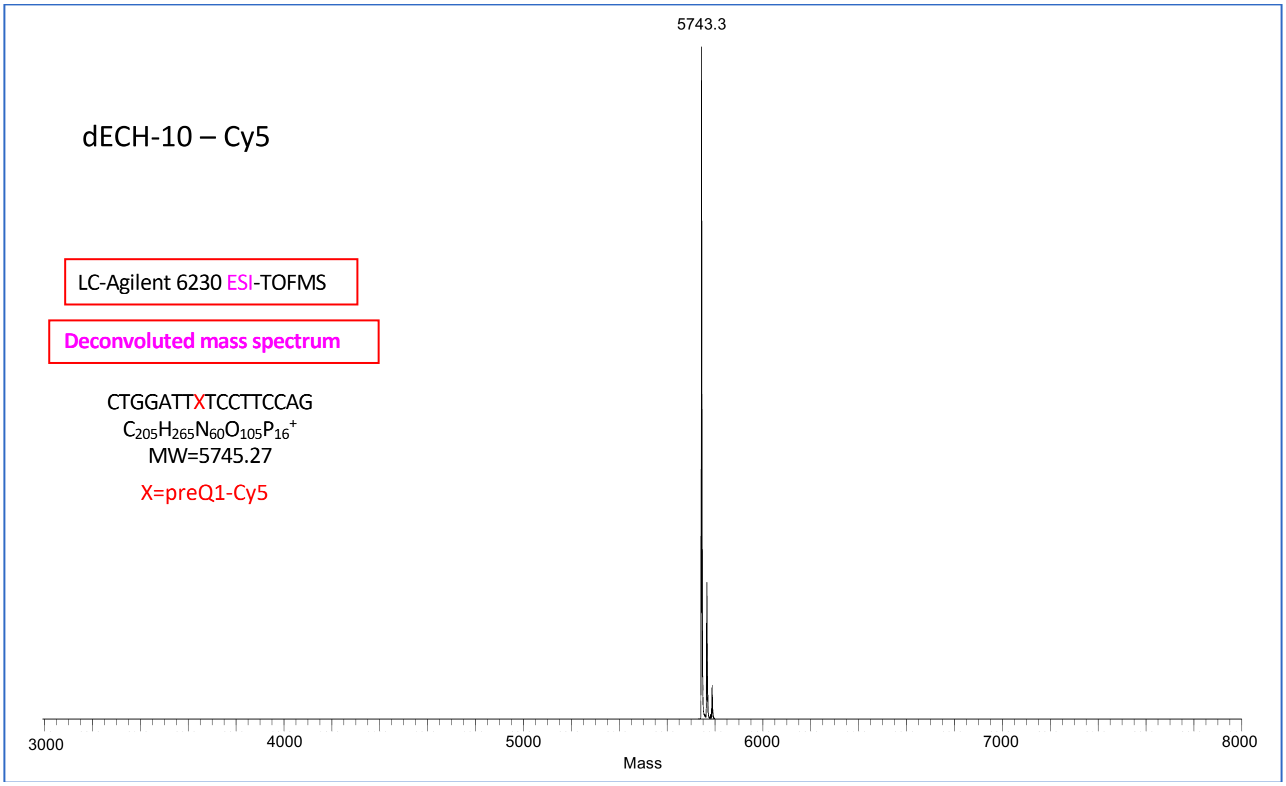
5743.3
dECH-10 – Cy5
LC-Agilent 6230 ESI-TOFMS
Deconvoluted mass spectrum
CTGGATTXTCCTTCCAG
$C_{205}H_{265}N_{60}O_{105}P_{16}^{+}$
MW=5745.27
X=preQ1-Cy5
3000
4000
5000
6000
7000
8000
Mass

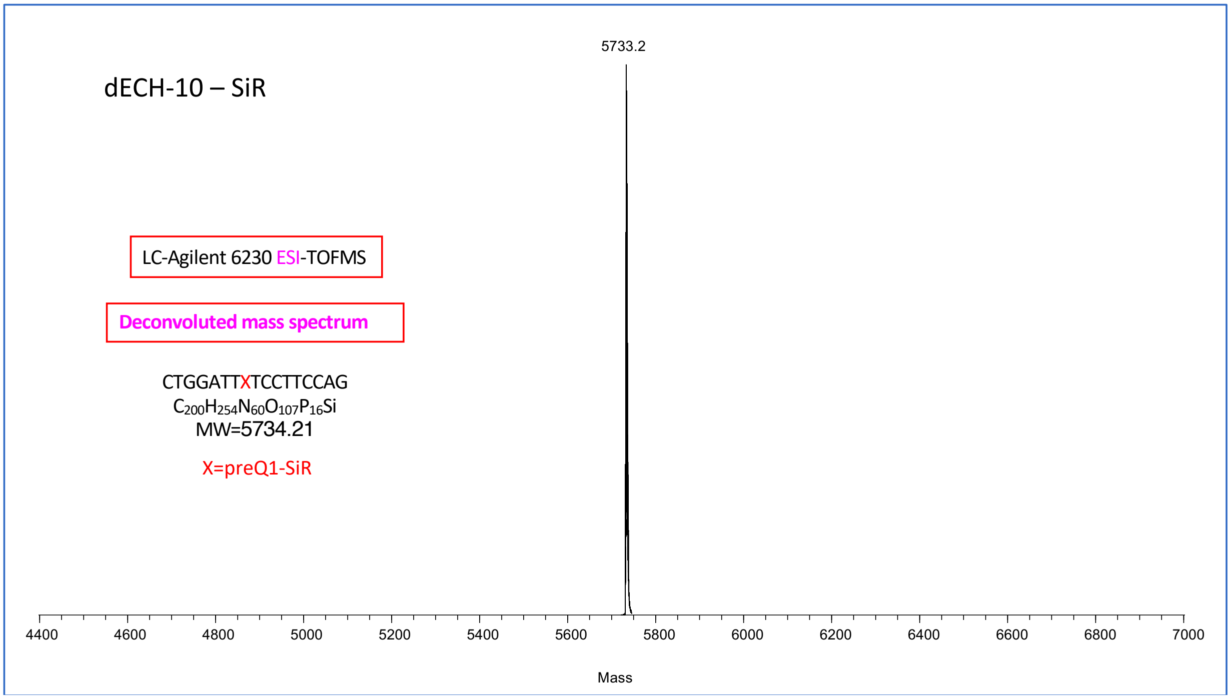
5733.2
dECH-10 – SiR
LC-Agilent 6230 ESI-TOFMS
Deconvoluted mass spectrum
CTGGATTXTCCTTCCAG
C200H254N60O107P16Si
MW=5734.21
X=preQ1-SiR
4400
4600
4800
5000
5200
5400
5600
5800
6000
6200
6400
6600
6800
7000
Mass

14. References

(1) Alexander, S. C.; Busby, K. N.; Cole, C. M.; Zhou, C. Y.; Devaraj, N. K. Site-Specific Covalent Labeling of RNA by Enzymatic Transglycosylation. *J. Am. Chem. Soc.* **2015**, *137* (40), 12756–12759.
(2) Busby, K. N. & Devaraj, N. K. Enzymatic covalent labeling of RNA with RNA transglycosylation at guanosine (RNA-TAG). *Meth. Enzymol.* **2020**, 641, 373-399.
